# A pivotal Wnt antagonist role promoting digit joint specification by constraining Wnt activity

**DOI:** 10.1038/s41467-026-73549-4

**Published:** 2026-05-26

**Authors:** Bau-Lin Huang, Sean Davis, Eiki Koyama, Maurizio Pacifici, Susan Mackem

**Affiliations:** 1 Cancer & Developmental Biology Laboratory, CCR, National Cancer Institute, Frederick, MD USA; 2 Genetics Branch, CCR, National Cancer Institute, Bethesda, MD USA; 3 Translational Research Program in Pediatric Orthopaedics,Division of Orthopaedic Surgery, Children’s Hospital of Philadelphia, Philadelphia, PA USA; 4 Present Address: Univ. Colorado-Anschutz Medical Campus, Denver, CO USA

**Keywords:** Morphogen signalling, Pattern formation, Morphogenesis

## Abstract

Bmps and Wnts often act antagonistically. Here we report that in mouse digit progenitors, unlike long bone joints, they cooperate to promote chondrogenic over joint (interzone) commitment. Elevated Bmp signaling prevents 5’*Hoxd*^Δ/Δ^ digit progenitors from forming interzones, causing joint loss. We show that constitutive βCatenin activation (βCatCA) in 5’*Hoxd*^Δ/Δ^ interdigits restores digit joints indirectly and cell non-autonomously. RNA profiling reveals βCatCA induces secreted Wnt antagonists that restore 5’*Hoxd*^Δ/Δ^ digit joints by reducing digit-tip Bmp activity. Deleting the βCatCA-induced Wnt antagonist *Dkk2* in 5’*Hoxd*^Δ/Δ^ interdigits abolishes joint rescue by βCatCA. Wnts inhibit Gsk3β kinase, which phosphorylates and destabilizes both βCatenin and Bmp-activated receptors pSmad1/5. In cultured limb buds, Gsk3β antagonists stabilize pSmad1/5, enhancing digit-tip Bmp activity. We propose that Wnt antagonists prevent precocious pSmad1/5 accumulation by stabilizing Gsk3β, favoring joint fate. Excess Bmp-pSmad1/5 activity in 5’*Hoxd*^Δ/Δ^ digit-tips accelerates chondrogenic commitment, impeding a switch to joint fate. Wnt antagonists maintain mesenchymal plasticity for normal phalanx-joint specification by slowing the pace of chondrogenic commitment.

## Abstract (web summary)

Wnts induce joints in long bone cartilage, but their role in digit joints which arise coordinately with phalanges is unclear. Here they show that Wnts act with Bmps to promote digit progenitor chondrogenic fate. Wnt antagonists maintain plasticity for joint/phalanx fates.

## Introduction

Joints are critical for skeletal mobility and functional limb adaptations, particularly in the hand/foot (autopod) region. The number of phalanges and joints serve as hallmarks of digit identity and vary over a wide range in tetrapod vertebrates through evolutionary adaption for different functions, such as flying, swimming, running^1^. Understanding the steps regulating phalanx and joint formation offers insights into how early limb patterning is linked to later events in skeletal morphogenesis at the molecular level, as well as illuminating the developmental basis for adaptive evolution. Manipulation of several major signaling pathways can alter the position and number of joints and phalanges in the developing digits^2–5^. Yet how these pathways coordinate in vivo to determine digit morphology and joint formation is still poorly understood.

Digit phalanx-forming progenitors arise following the formation of metapodial condensations (metacarpals/metatarsals), or “digit rays” in amniotes, and contribute to a phalanx-forming region (PFR) that first becomes evident under the Fgf-producing apical ridge (AER) just distal to the metapodial rays at about E12-12.5 in mouse^1,6,7^. In both chick and mouse, progenitors in the digit tip are regulated independently of metapodial rays and respond to adjacent signaling that determines their potential for coordinate phalanx and interzone formation and final digit “identity” (numbers of phalanges/joints formed)^6,8^. Bmp-, Wnt- and Fgf- are major pathways active at the onset of phalanx-forming stages. The interplay of Fgfs and canonical Wnts maintain mesenchymal progenitors in an undifferentiated and proliferative state that is poised to assume a chondrogenic fate. However, exposure of the mesenchymal progenitors to Wnts in the absence of Fgfs results in stable epigenic changes that suppress *Sox9* activation and chondrogenic fate^9,10^. Initiation of a PFR-organizing center in the digit tip involves convergent-extension tissue flows induced by noncanonical Wnt activity, and Bmps are required to promote compaction of limb mesenchymal cells prior to Sox9 expression as well as during formation of discrete Sox9+ chondrogenic condensations^11–13^.

Both interzone (joint precursor) cells and chondrocytes are derived from common mesenchymal Sox9+ pre-chondrogenic cells^14^. However, in contrast to long bones where joints arise within pre-existing cartilage anlage, digit joint and phalanx precursors are specified coordinately in distal tip progenitors transiting into PFR to give rise to sequential phalanx-interzone anlage in response to interdigit Bmp signaling and local Bmp down-modulation by Noggin^3,6,8,15^. In both chick and mouse, the net Bmp activity level in different interdigits is thought to instruct the pace of periodic phalanx formation in the adjacent PFR, resulting in different phalanx numbers and digit identities^6,8^. Furthermore lineage tracing of digit tip cells in chick, using a micro-injected defective retroviral reporter to selectively mark cell populations, unequivocally showed that new phalanges arise as discrete elements from a distinct, sub-AER progenitor pool, and not as an extension from pre-existing proximal Sox9+ chondrogenic elements. Based on the initial appearance of sequential *Gdf5*+ interzones from the distalmost border of the digit tip, it is presumed that this process is conserved in mouse^6^, but definitive lineage tracing of the progenitors is not yet possible in the absence of distinctive molecular markers to enable selective genetic tagging of progenitors in vivo.

Interzone specification entails a loss of *Sox9* RNA and the initiation of *Gdf5* expression^14,16–18^, which serves as the earliest marker of interzone fate. Analysis of mouse mutants, as well as manipulation of signaling in chick, has identified both canonical Wnt^4,19^ and Bmp^2,20,21^ pathways as playing pivotal roles in directing this process during long bone development, but the role of Wnts in digit joint formation is less clear. Even in the context of long bone joints, although Wnt/βCatenin activation induces ectopic *Gdf5* expression, β*Catenin* removal in early mouse limb bud does not alter the onset of interzone *Gdf5* expression^22^, suggesting that events upstream of Wnt activation may direct initial interzone specification.

In this report, to delineate the role of Wnt/βCatenin in interzone specification in developing digits, we selectively express a stabilized (constitutively-active), conditional β*Catenin* allele (β*Catenin*^*+/Exon3Flox*^, hereafter referred to as βCatCA)^23^ either in 5’*Hoxd*^Δ/Δ^ phalangeal condensations or interdigit tissue. Unexpectedly we find that βCatCA, acting cell non-autonomously when expressed in interdigit mesenchyme, restores joint formation by inducing expression of Wnt antagonists that counteract elevated Bmp-pSmad1/5 activity in the 5’*Hoxd*^Δ*/*Δ^ distal digit tip. In contrast to previous work supporting opposing roles of Bmps and Wnts in long bone joint formation, we conclude that canonical Wnts cooperate with Bmps to impede interzone specification and that Wnt antagonists play a pivotal early role in constraining Wnt activity to maintain the plasticity of cells transiting into nascent PFR, allowing them to adopt either chondrogenic or interzone fates. We propose that canonical Wnts cooperate with Bmps by inhibiting Gsk3β and thereby stabilizing pSmad1/5, which in turn accelerates the transition to chondrogenic commitment and impairs a switch to interzone fate in digit tip progenitors.

## Results

### Interdigit βCatenin activation restores joints in 5’Hoxd^∆/∆^ digits

**Fig. 1:**
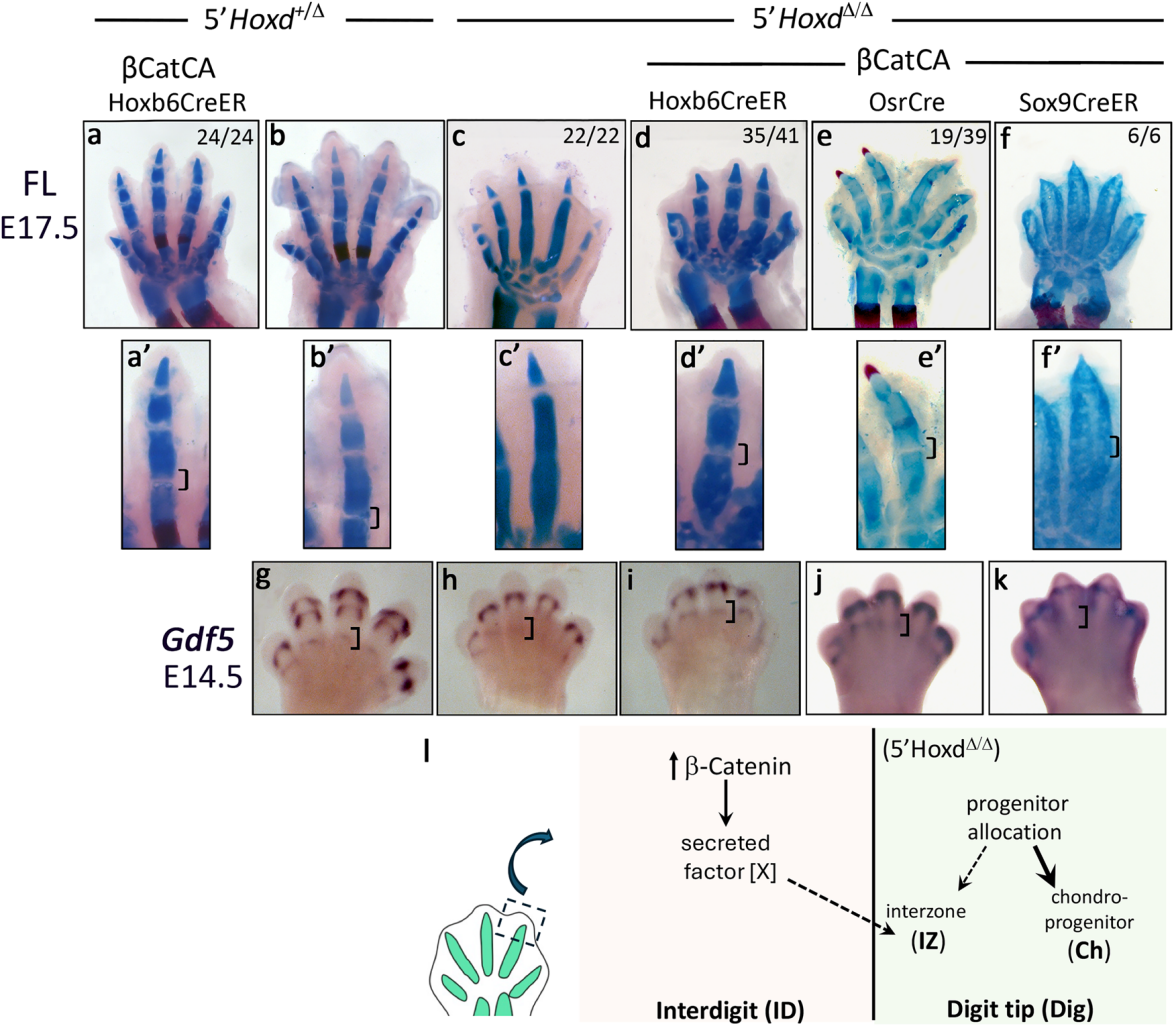
Digit joint formation restored in 5’*Hoxd*^Δ/Δ^ by βCatCA-activation in interdigits with Hoxb6CreER or OsrCre. **a–f** E17.5 limb skeletons show digit morphology either with (a, d–f) or without (b, c) the particular Cre- or CreER-activated βCatCA, as indicated, in 5’*Hoxd*^Δ/Δ^ and control (*Hoxd*^+/Δ^) siblings. **a’–f’** Enlarged images show joint formation in digit 3 for each genotype. Bracket indicates the presumptive position of metacarpal-phalangeal (MCP) joint. The numbers in the images indicate the frequency of the observed phenotype shown in limbs analyzed for each genotype indicated and collected from 6 independent litters for (a–d**)**, seven litters for (e), and three litters for (f). **g–k** in situ hybridization shows *Gdf5* expression in E14.5 interzones/early joints. Bracket indicates presumptive position of MCP interzone. Two independent litters analyzed for *Gdf5* including two limbs per genotype indicated and giving same results as shown for each litter. **l** Schematic summarizing findings that (ID)-activated βCatCA acts cell non-autonomously on digit tip (Dig) progenitors to enhance interzone specification by affecting some step in commitment of progenitors to chondrogenic (Ch) or interzone (IZ) fate. Tamoxifen was injected at E11.25 (3 mg dose per pregnant dam) for Hoxb6CreER and for Sox9CreER.

*5’Hoxd* (*Hoxd11, d12, d13*) gene deletion (5’*Hoxd*^Δ/Δ^) results in brachydactyly (bi-phalangeal digits) of all digits and loss of metacarpal-phalangeal joints in forelimb digits 3 and 4 (100%), with lower penetrance in digits 2 and 5 (15%–30% frequency)^6,24,25^ compared to 5’*Hoxd*^+/Δ^ controls (Fig. 1a–c). We previously showed that reducing Bmp activity in *5’Hoxd*^Δ/Δ^ autopod restores phalanx and digit joint formation^6^. We also evaluated if canonical Wnt activity could rescue interzone formation using βCatCA. Unexpectedly, selective βCatCA activation in digit chondroprogenitors (by Sox9CreER) did not restore joint formation in 5’*Hoxd*^Δ/Δ^ digits^6^, contrary to expectations based on prior work showing that canonical Wnt activity promotes joint formation in long bones by antagonizing Sox9 and chondrocyte differentiation^19,26,27^. This result led us to ask whether canonical Wnt signaling plays any role in digit joint formation analogous to that in long bone joint induction.

To confirm and extend our previous results, we compared the effects of expressing conditional βCatCA selectively in complementary limb bud regions of 5’*Hoxd*^Δ/Δ^ embryos, using either Sox9CreER or Hoxb6CreER to activate βCatCA expression prior to the onset of digit phalanx/interzone specification (Tamoxifen at E11.25). In contrast to Sox9CreER, Hoxb6CreER is expressed throughout undifferentiated limb mesenchyme and shuts off in cells beginning to undergo chondrogenic differentiation^28,29^. As previously reported, chondrogenesis was severely impaired in 5’*Hoxd*^Δ/Δ^ digits by Sox9CreER-activated βCatCA, but there was no evidence of restored interzone formation, based either on morphology or *Gdf5* expression (*n* = 6/6, compare Fig. 1f, k to c, h). In contrast, interdigit βCatCA activation by Hoxb6CreER efficiently restored metacarpal-phalangeal (MCP) joint formation in digits 3 and 4 of 5’*Hoxd*^Δ/Δ^;βCatCA embryos, both at the level of interzone *Gdf5* expression and subsequent skeletal morphology, (*n* = 35/41, Fig. 1d, i). However, phalanx restoration (to triphalangeal digits) was never seen (*n* = 35/35). βCatCA had negligible phenotypic effects on sibling 5’*Hoxd*^+/Δ^;βCatCA controls, except for loss of one phalanx in digit 5 (*n* = 24/24, compare Fig. 1a, b).

Time-course analysis for effective rescue by Hoxb6CreER-activated interdigit βCatCA indicated that rescue efficiency declined rapidly when Tamoxifen was injected later than E11.25 and rescue was lost by E11.75 (Supplementary Fig. 1). Inclusion of a Rosa-tdTomato reporter confirmed that Hoxb6CreER-activated βCatCA targeted predominantly the interdigit mesenchyme, with scarce descendant recombinant cells subsequently present in mature cartilage at late stages (E14.5, E16.5), and with no contribution to the restored digit joint regions (Supplementary Fig. 2a, b). To rule out rescue by scarce βCatCA-expressing chondrogenic cells contributing to interzone directly, we used a highly restricted, interdigit-specific OsrCre transgene^30^ to activate βCatCA solely in interdigits. 5’*Hoxd*^Δ/Δ^ joint formation was indeed restored by OsrCre-activated βCatCA expression, albeit only in digit 3 (*n* = 19/39, Fig. 1e, j). Owing to the late onset of OsrCre expression in different interdigits (restricted to interdigits adjacent to d2-d3 at E12.5 and only later extending to d4-d5 by E13.5^30^, see Supplementary Fig. 2b), OsrCre-driven βCatCA activity only restored joint formation in digit 3 and with lower frequency than seen following Hoxb6CreER-driven βCatCA activation. Rosa-tdTomato lineage analysis indicated that OsrCre-activated βCatCA-descendent cells in 5’*Hoxd*^Δ/Δ^; βCatCA limbs remained completely restricted to interdigit tissue even at late skeletal stages (E16.5) and no labeled cells were observed in cartilage or interzone regions (Supplementary Fig. 2c). Together, these results indicate that βCatCA acts indirectly from interdigit tissue to promote interzone fate (Fig. 1i).

Joint formation is a multi-step process, that begins with induction of interzone *Gdf5* expression, and eventually leads to cavitated, mature joints. To determine if early-stage 5’*Hoxd*^Δ/Δ^;βCatCA rescued joints later progress to mature joint formation, we examined the late-stage expression of several joint developmental and maturation markers (*Gdf5, Wnt9a, Proteoglycan 4* (*Prg4*), *Col IIA;* Supplementary Fig. 3). Whereas these genes were barely expressed in 5’*Hoxd*^Δ/Δ^ prospective joint sites, they were all expressed comparably to sibling controls between E14.5 and P0 in joints restored by OsrCre-activated βCatCA (Supplementary Fig. 3a, b). Full digit joint cavitation was also restored by P0 (Supplementary Fig. 3b), indicating that the rescued joints persist and mature.

Together, these results indicate that βCatCA, acting indirectly from interdigits, restores interzone formation in digit tip progenitors. Both the early time requirement for efficient interzone restoration by Hoxb6CreER (tamoxifen at E11.25) and the lower efficacy of OsrCre-driven βCatCA activation, which is expressed later and in a limited region at E12.5, also suggest an early requirement for interdigit βCatCA activity at the time of initial interzone induction, rather than at later interzone maturation stages.

### Time frame of digit tip progenitor appearance and commitment

**Fig. 2:**
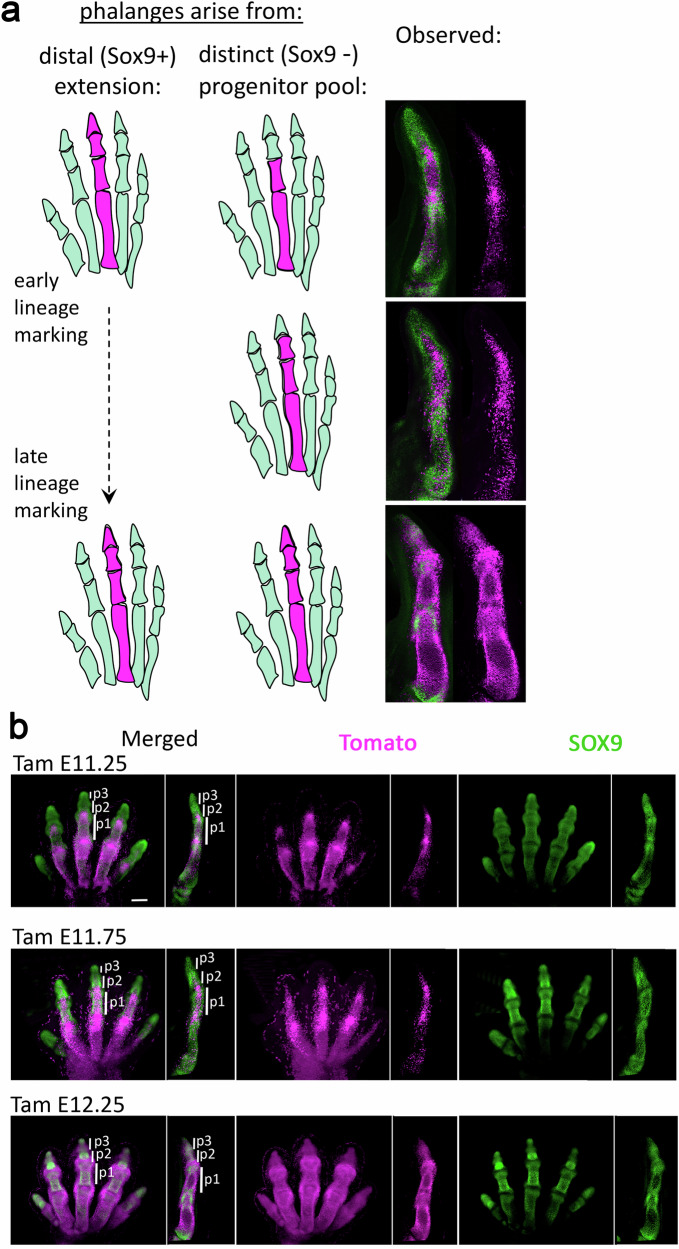
*Sox9*+ lineage marking at stages ranging from onset to termination of phalanx induction show that new phalanx progenitors do not arise from pre-existing Sox9+ elements/PFR. Tamoxifen injection at different times indicated in panel (b) and extent of Cre-reporter Rosa-Tomato+ cells (pseudocolored magenta) contributing to digits evaluated at E13.5 with confocal imaging (Sox9 immunofluorescence green). **a** schematic of expected outcomes if new phalanges arise from distal end of existing Sox9+ PFR/phalanges vs distinct distal progenitor pool separate from committed PFR region. **b** results of Tomato-reporter marking at E11.25 prior to P1 anlage, E11.75 prior to P2 anlage, or E12.25 prior to P3 anlage appearance are shown for both antero-posterior (full handplate) and dorso-ventral (digit 3) views. Enlarged optical section of individual digit 3 result also shown in (a) schematic. For each time point indicated, four embryos (eight limbs) were analyzed from at least two separate litters with same results as shown. Tamoxifen was injected at reduced dosage (of 0.05 mg per pregnant dam) to limit the duration of effective in vivo levels. p1-p3, phalanges 1–3. Scale Bar in (b), 200 μm for all panels in (b).

The time window during which tamoxifen-activated βCatCA efficiently restores 5’*Hoxd*^Δ/Δ^ interzone fate is limited to an early stage when digit tips and PFR first appear. This prompted us to examine more closely the timing of progenitor appearance and commitment to phalanx formation in mouse relative to when new interzones appear. We previously showed that new digit interzones, detected by *Gdf5* expression, first emerge sequentially from the very distal edge of the digit tip starting at about E12.25 and become displaced proximally over time, rather than arising within a pre-formed condensation^6^. This suggests that phalanges in mouse arise from distal tip progenitors in a manner similar to chick, but does not by itself exclude the possibility that new phalangeal elements arise by distal extension from existing phalanges (see Fig. 2a).

In chick, lineage tracing of the sub-AER (progenitor) zone labels only distal phalanges that have not yet begun to form at the time of marking, whereas lineage tracing of the more proximal Sox9+ PFR zone labels only the proximal, already complete and forming phalanges, but not distal elements yet to arise^8^. In mouse, definitive lineage tracing of progenitors is not possible owing to the lack of known markers to selectively tag these cells in vivo. However, using Sox9CreER and timed tamoxifen injections, we find that, as in chick, sequential phalanges in mouse digits do not arise by distal extension from existing proximal, Sox9+ chondrogenic anlage. Rosa-tdTomato reporter activation by tamoxifen-induced Sox9CreER at different times reveals that early Sox9CreER activation (E11.25) marks only proximal phalanges; marking of more distal phalanges occurs sequentially following Cre-activation at progressively later times (tamoxifen at E11.75 or at E12.25; Fig. 2b). Together with the initial appearance of sequential *Gdf5*+ bands from the very distal digit tip, this supports a model in which nascent interzone-phalanx fates arise together in distal tip progenitor cells newly contributing to PFR.

### Onset of 5’Hoxd^∆/∆^ digit tip phenotype coincides with interzone induction

**Fig. 3:**
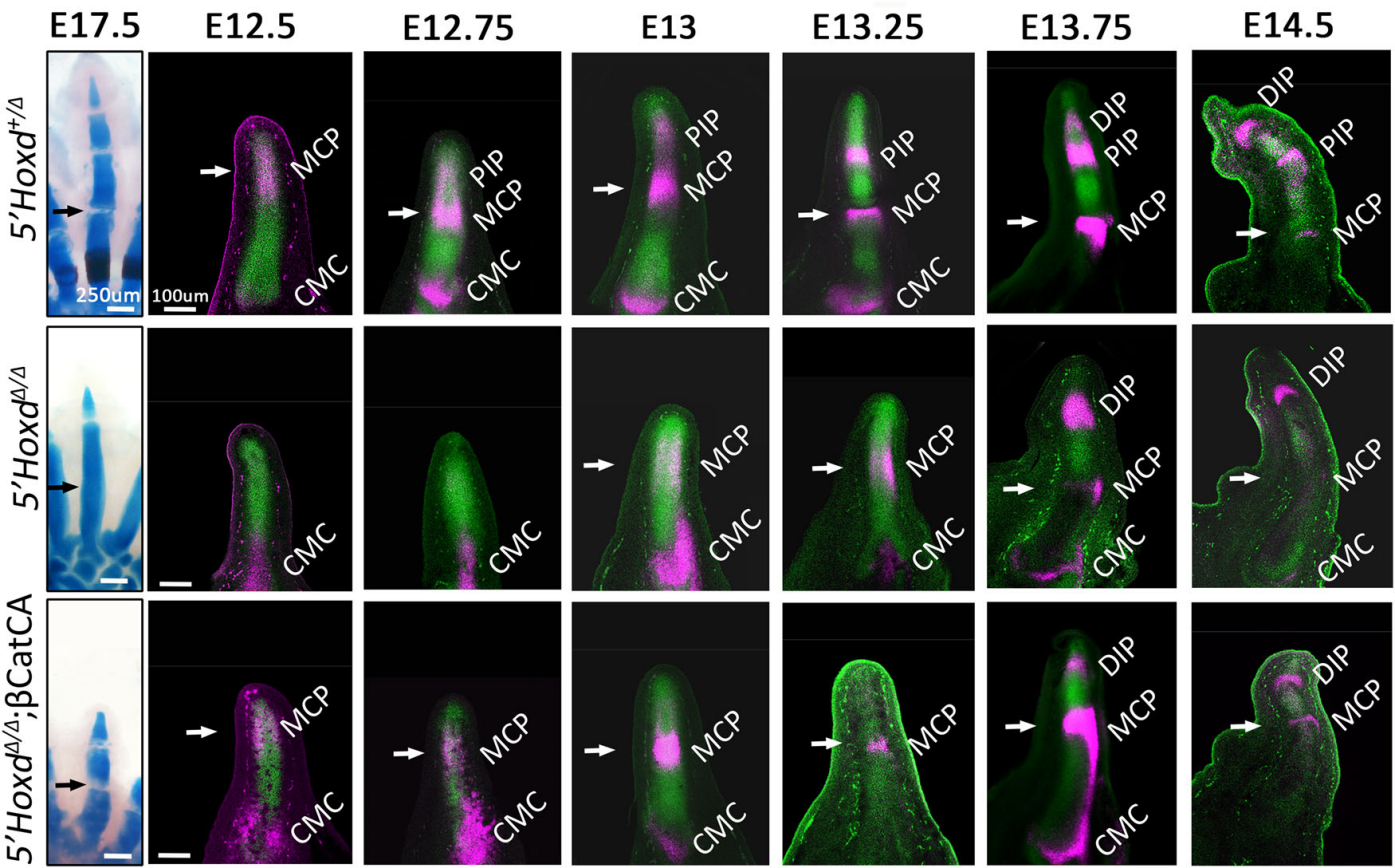
Time course of sequential appearance of *Gdf5*+ interzones during phalanx induction. HCR in situ staining for *Gdf5* (magenta) and *Sox9* (green) in 5’*Hoxd*^+/Δ^, 5’*Hoxd*^Δ/Δ^ and 5’*Hoxd*^Δ/Δ^;βCatCA digit tips. Panels at left show mature (E17.5) skeletal stained phalanges of digit 3 for comparison. In 5’*Hoxd*^+/Δ^ controls, sequential *Gdf5*+ interzones emerge from the distal digit tip border in the order of MCP (metacarpal-phalangeal), PIP (proximal interphalangeal), and DIP (distal interphalangeal) interzone. A clear *Gdf5* + MCP interzone fails to appear in 5’*Hoxd*^Δ/Δ^, but a transient, external *Gdf5+* ring appears and later disappears. The PIP is absent, but DIP forms at normal time and position. In 5’*Hoxd*^Δ/Δ^;βCatCA digit tips, the *Gdf5* + MCP interzone appears with the same timing as in controls. Arrows indicate MCP interzone, or joint (at E17.5). Scale bar of 100 μm, shown for E12.5, applies to all E12.5-E14.5 images. Scale bar of 250 μm shown for all E17.5 skeletal panels. CMC, carpo-metacarpal (wrist) interzone. For each embryonic age evaluated, four limbs of each genotype as indicated were analyzed for *Gdf5* in at least two separate experiments with same results as shown.

To determine the earliest time of detectable 5’*Hoxd*^Δ/Δ^ interzone phenotypes and assess when restoration of interzone fate by interdigit βCatCA first becomes evident, we examined the time course of sequential *Gdf5*+ interzone appearance in control compared to 5’*Hoxd* mutant and rescued mutant digits. As shown in Fig. 3 and Supplementary Fig. 4, *Gdf5* first appears distally in controls at about E12.5, marking the proximal metacarpo-phalangeal (MCP) interzone, followed by the proximal interphalangeal interzone (PIP), which first appears in distal tip by E12.75 and becomes a clear band by E13, delimiting the P1 condensation. In 5’*Hoxd*^*+*/Δ^ controls this is followed by appearance of the distal interphalangeal (DIP) interzone, apparent by E13.75, delimiting the P2 condensation (Fig. 3, Supplementary Fig. 4). The 5’*Hoxd*^Δ/Δ^ MCP and PIP interzones fail to appear but the DIP interzone is clearly evident at the same time that it emerges in controls (Fig. 3). In the βCatCA-rescued 5’*Hoxd* mutant, the MCP *Gdf5*+ interzone first appears at E12.5, very similar to controls, but because the P2 phalanx is still absent (digits are still bi-phalangeal), no PIP interzone forms. The DIP interzone again appears in βCatCA-rescued 5’*Hoxd*^Δ/Δ^ with similar timing as both control and 5’*Hoxd* mutant digits, despite the loss of P2.

Notably, a peripheral *Gdf5*+ band was observed transiently around the prospective MCP interzone region in 5’*Hoxd* mutants (Fig. 3). A similar circumferential ring of *Gdf5*-expressing cells has been seen previously in other mutants with joint loss phenotypes, including *Creb5*, *Noggin* or *Indian hedgehog* (*Ihh*) null mutants^2,31,32^, suggesting an independent regulation of external/interdigital and interzone *Gdf5* expression.

The normal appearance of the initial *Gdf5* + MCP interzone by E12.5 in control and in rescued 5’*Hoxd* mutants, together with the timing of tamoxifen-induced βCatCA activation required for effective rescue, and similar timing of progenitor commitment to P1 phalanx formation (E11.25-E11.5, Fig. 2, Supplementary Fig. 1), point to an early-stage interzone induction phenotype in the 5’*Hoxd* mutant beginning at E12-12.5 (taking into account a ~ 12–18 h delay in tamoxifen-activated reporter expression^33^). Accordingly, we focused our subsequent analyses of gene expression in the 5’*Hoxd*^Δ/Δ^ and rescued mutant on E12.5.

### Interdigit βCatCA reduces Bmp activity in 5’Hoxd^∆/∆^ digit tip progenitors

**Fig. 4:**
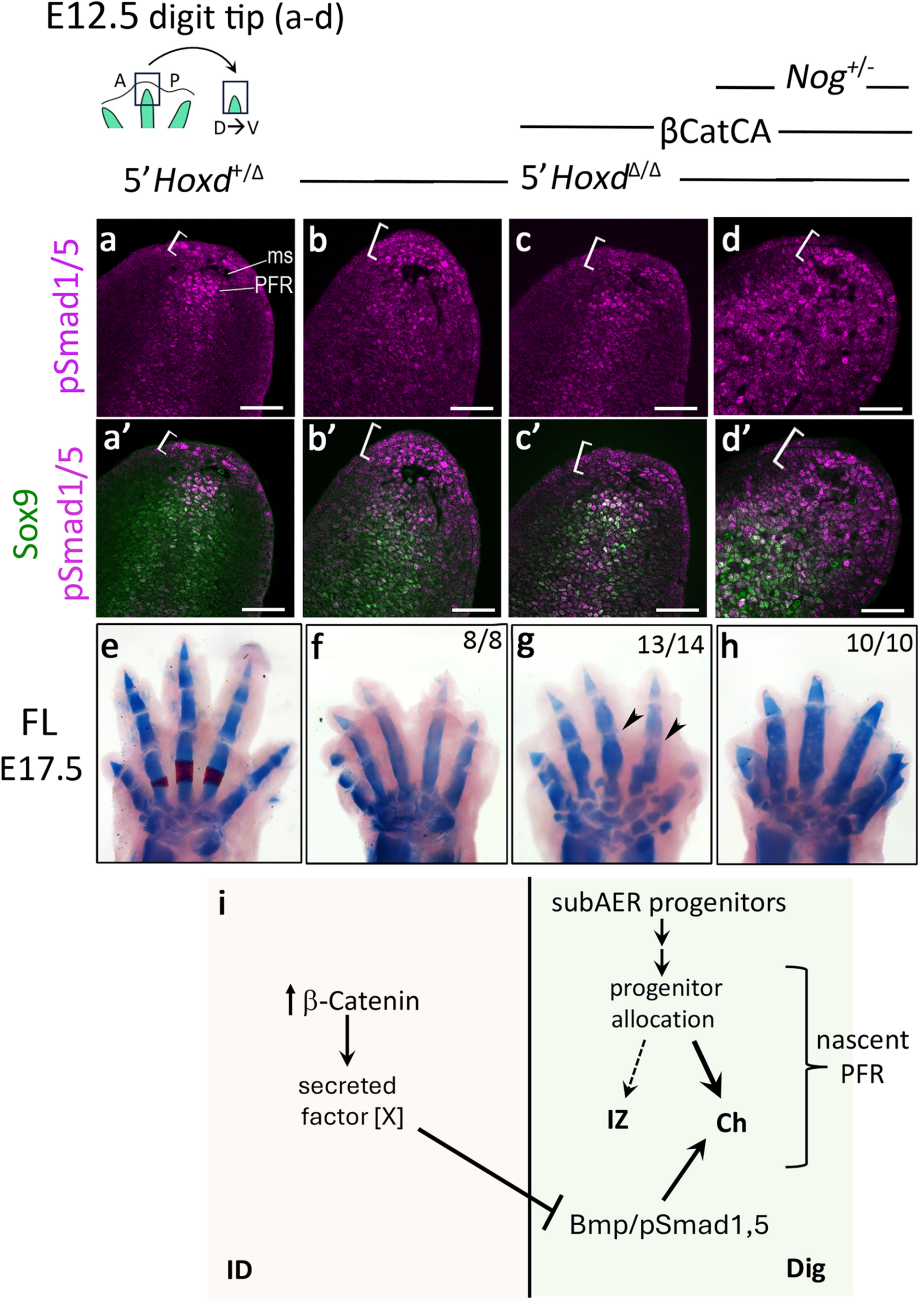
βCatCA activation in 5’*Hoxd*^Δ/Δ^ interdigits acts by reducing Bmp activity in the digit tip. **a–d** pSmad1/5 staining of E12.5 distal digit tip sections (dorsoventral, shown in schematic) in βCatCA-rescued 5’*Hoxd*^Δ/Δ^ and effect of reducing the Bmp-antagonist *Noggin* gene dosage (*Nog*^+/-^) on rescue by βCatCA. Interdigit Hoxb6CreER-activated βCatCA, 3 mg tamoxifen injection at E11.25. Brackets indicate sub-AER mesenchyme; ms, marginal sinus capillaries; PFR, phalanx forming region. Two experiments performed, each including two limbs for each genotype indicated, that gave the same results. **a’–d’** merged images of pSmad1/5 and Sox9 immunofluorescence. Scale Bar, 50 μm in all panels. **e–h** E17.5 skeletal stain showing subsequent effects on mature digit joint formation. Arrowheads indicate MCP digit joints rescued by interdigit βCatCA activation in 5’*Hoxd*^Δ/Δ^ and loss of rescue by reducing *Noggin* in 5’*Hoxd*^Δ/Δ^;βCatCA (compare panels (g) and (h)). Numbers in panels indicate frequency of phenotype shown in total limbs analyzed for each genotype indicated and collected from six independent litters. **i** schematic summarizing findings that interdigit (ID)-activated βCatCA restores digit interzone specification indirectly (cell non-autonomously; see Fig. 1) and correlates with reduced Bmp activity-pSmad1/5 in 5’*Hoxd*^Δ/Δ^;βCatCA digit tips. Reducing *Noggin* dosage abolishes the pSmad1/5 reduction and subsequent joint rescue, suggesting that excess Bmp-pSmad1/5 activity accelerates the pace of chondrogenic (Ch) vs interzone (IZ) commitment in digit tips (Dig) and impairs interzone fate.

Previous work in chick implicated interdigit signals as key regulators that differentially instruct digit identity (final phalanx/joint number) of adjacent digit tip progenitors contributing to PFR^3,8,34^. We previously showed that digit tip cells can adopt either a chondrogenic or joint fate depending on their level of Bmp response and that elevated endogenous Bmp activity present in the *5’Hoxd*^Δ/Δ^ digit tip inhibits interzone formation^6^. To determine if βCatCA acts indirectly to restore *5’Hoxd*^Δ/Δ^ interzone formation by down-regulating Bmp response, we compared immunofluorescence intensity of pSmad1/5 in 5’*Hoxd*^Δ/Δ^ and βCatCA-rescued E12.5 digit tips. As shown previously^6^, 5’*Hoxd*^Δ/Δ^ digit tip-pSmad1/5 staining was elevated compared to sibling controls, particularly in the sub-AER mesenchyme as well as in PFR (Fig. 4b compared to 4a, brackets), and was reduced in 5’*Hoxd*^Δ/Δ^;βCatCA embryos at E12.5 (Fig. 4c, bracket). Activation of pSmad3 by ActivinβA is thought to act upstream of Bmp-pSmad1/5 signaling in the PFR^13,35^ and we also compared the levels of *ActivinβA* and pSmad3 in 5’*Hoxd*^Δ/Δ^ with the βCatCA-rescued mutant. Unlike pSmad1/5, both *ActivinβA* expression (Supplementary Fig. 5a) and pSmad3 activity (Supplementary Fig. 5b) appeared unaltered in either the 5’*Hoxd* mutant or rescued mutant compared to sibling controls, suggesting that the effect of βCatCA-rescue on the 5’*Hoxd* mutant occurs primarily at the level of Bmp-pSmad1/5 signaling.

To confirm that interdigit βCatCA restored joint formation by reducing Bmp signaling to the digit tip, we examined the effect of increased Bmp levels on the rescue by reducing gene dosage of the Bmp antagonist *Noggin*. Immunodetectable pSmad1/5 in 5’*Hoxd*^Δ/Δ^;βCatCA;*Nog*^*+/-*^ sub-AER (bracket) and PFR regions was much higher than in 5’*Hoxd*^Δ/Δ^;βCatCA (Fig. 4d compared to 4c) and more similar to 5’*Hoxd* mutant alone (Fig. 4b), and the subsequent rescue of digit joint formation was completely abolished (compare digits 3, 4 in Fig. 4 g, arrows, *n* = 13/14 with joint rescue, and in 4 h, *n *= 10/10 with no rescue). Thus interdigit βCatCA restores digit joint formation by down-regulating the Bmp response in digit tips indirectly (Fig. 4i).

### Interdigit RNAseq identifies candidates for joint rescue in 5’Hoxd^∆/∆^;βCatCA

**Fig. 5:**
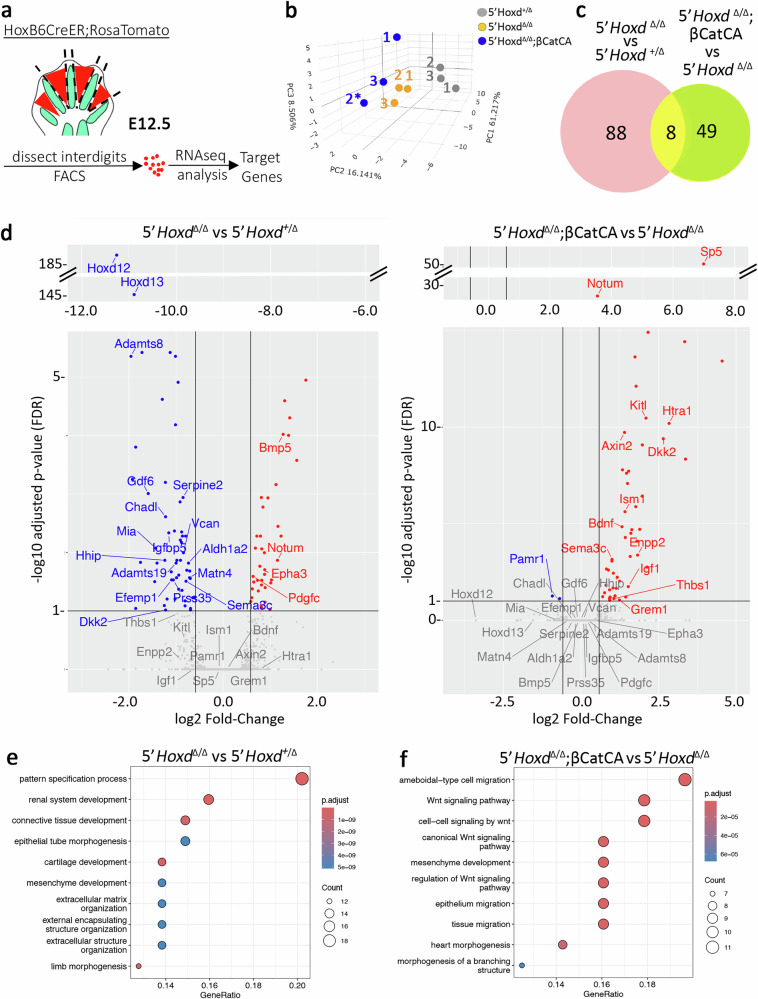
Transcriptome analysis identifies Wnt antagonists as candidate genes for restoring digit joint formation in 5’*Hoxd*^Δ/Δ^;βCatCA. **a** Experimental design of interdigit tissue isolation for transcriptome analysis. Interdigits between digits 2-3, 3-4 and 4-5 dissected and pooled, dissociated and FACS sorted. βCatCA activated by Hoxb6CeER with 3 mg Tamoxifen injection at E11.25. **b** 3D-PCA analysis of normalized gene expression for complete transcriptome (in Supplementary data set 2) shows distinct segregation of each genotype with three biological replicates. (see also Supplementary information for replicate 1–3 annotation and Supplementary Fig. 6 for 2D-PCA plots). **c** Venn diagram shows 8 out of 145 total significant DE genes that are shared between the 5’*Hoxd*^Δ/Δ^ vs 5’*Hoxd*^+/Δ^ and 5’*Hoxd*^Δ/Δ^;βCatCA vs 5’*Hoxd*^Δ/Δ^ comparisons (FC ≥ 1.5; FDR ≤ 0.1) tabulated in Supplementary data set 1. **d** Volcano plots visualizing genes in (c) are shown in red for positive DE, blue for negative DE, and gray for non-significant DE in the 5’*Hoxd*^Δ/Δ^ vs 5’*Hoxd*^+/Δ^ or 5’*Hoxd*^Δ/Δ^;βCatCA vs 5’*Hoxd*^Δ/Δ^ comparisons (FC ≥ 1.5; FDR ≤ 0.1). Volcano plots generated from DEseq2 analysis using default parameters with 2-tailed test as detailed in methods and tabulated in Supplementary data set 1. See also heatmaps in Supplementary Fig. 7. Relevant DE extracellular factors are annotated as well as *Axin2, Sp5* expected to be highly induced by βCatCA and *Hoxd13*, *Hoxd12* expected to be absent in 5’*Hoxd*^Δ/Δ^. **e** Gene-set GO biological process enrichment for the 96 DE genes in 5’*Hoxd*^Δ/Δ^ vs 5’*Hoxd*^+/Δ^ comparison. **f** Gene-set GO biological process enrichment for the 57 DE genes in 5’*Hoxd*^Δ/Δ^;βCatCA vs 5’*Hoxd*^Δ/Δ^ comparison. GO biological process enrichment of DE genes listed in Supplementary data set 1 performed using clusterProfiler R package version 4.10.0^44^ and results adjusted for significance based on Benjamini-Hochberg with a Q value cutoff of 0.01, and otherwise using default parameters.

The indirect, cell non-autonomous effects of interdigit βCatCA suggested that βCatCA may induce secreted targets that act on digit tip during interzone specification. To identify possible candidates acting downstream of βCatCA activity to restore 5’*Hoxd*^Δ/Δ^ digit interzones, we isolated FACS-sorted interdigit cells for RNAseq analysis comparing differential expression (DE) between 5’*Hoxd*^*+*/Δ^ control, 5’*Hoxd*^Δ/Δ^ and 5’*Hoxd*^Δ/Δ^;βCatCA sibling embryos. Because of its high rescue efficiency (near 100%), Hoxb6CreER was used to activate βCatCA, together with a Rosa-tdTomato reporter for FACS to isolate βCatCA-activated mesenchymal cells from dissected interdigits (Fig. 5a). Principal component analysis (PCA) showed that biological replicates for each genotype clustered separately from other genotypes (Fig. 5b, Supplementary Fig. 6). Notably, the 5’*Hoxd*^Δ/Δ^ and 5’*Hoxd*^Δ/Δ^;βCatCA replicates clustered more closely with each other than with 5’*Hoxd*^*+*/Δ^ controls, suggesting that interdigit βCatCA altered the expression of only a limited number of genes in the 5’*Hoxd*^Δ/Δ^ mutant background, consistent with DE analysis results (Supplementary Data files 1, 2 and summarized in Fig. 5c–f and Supplementary Fig. 7).

Comparisons between 5’*Hoxd*^Δ/Δ^ versus 5’*Hoxd*^*+/*Δ^ and 5’*Hoxd*^Δ/Δ^;βCatCA versus 5’*Hoxd*^Δ/Δ^ identified a total of 145 DE genes (FC > 1.5; Padj (FDR) < 0.1 filters), including 96 in 5’*Hoxd*^Δ/Δ^ vs 5’*Hoxd*^*+*/Δ^, 57 in 5’*Hoxd*^Δ/Δ^;βCatCA vs 5’*Hoxd*^Δ/Δ^, and 8 DE genes common in both comparisons (Fig. 5c, d Table 1, Supplementary Fig. 7 and Data Set 1). In agreement with several previous genetic and profiling studies^6,36,37^, Bmp pathway components were upregulated (e.g., *Msx2*; *Bmp5*), *Hoxa13* displayed compensatory upregulation upon *Hoxd13* loss, and several DE genes identified previously in E12.5 5’*Hoxd*^Δ/Δ^ autopod microarrays (*Papss2*, *Aldh1a2*, *Nr2f1* down-regulated; *Epha3, Tenm4/Odz4* up-regulated) behaved comparably in our DE analysis of 5’*Hoxd*^Δ/Δ^ vs 5’*Hoxd*^*+*/Δ^ (Supplementary Fig. 7 and Data sets 1, 2).

**Table 1:** Secreted factors differentially expressed in βCatCA rescue^a^

Gene	FC in 5’Hoxd^∆/∆^ vs 5’Hoxd^+/∆^:	p-adj:	FC in 5’Hoxd^∆/∆^;βCatCA vs 5’Hoxd^∆/∆^:	p-adj:
**Dkk2**	**-2.3**	0.09	**+6.3**	4e-10
**Notum**	+**2.2**	0.01	**+11.6**	2.4e-30
**Sema3c**	**−1.7**	0.03	**+2.0**	6.9e-4
**Bdnf**	NS‡	NS	**+2.5**	1.4e-5
**Enpp2**	−1.9	NS	**+3.5**	4.2e-4
**Grem1**	+1.2	NS	**+2.3**	0.09
**Htr4a1**	+1.7	NS	**+7.0**	6.4e-11
**Igf1**	+1.5	NS	**+2.8**	0.02
**Ism1**	NS	NS	**+2.6**	2.4e-6
**Kitl**	−1.7	NS	**+4.3**	3.4e-11
**Pamr1**	−1.6	NS	**-1.9**	0.06
**Thbs1**	−2.0	NS	**+2.1**	0.08

^a^Table 1 data from Supplementary data set 1; DEseq2 analysis of RNAseq data using

default parameters including 2-tailed hypothesis test (see details in Methods).

‡*NS* non-significant, *FC* Fold change, *p-adj* adjusted p value.

Genes that were DE in both comparisons were of particular interest, considering that any factor in this group with a normal role in interzone specification and also affected by βCatCA should be regulated oppositely in the 5’*Hoxd*^Δ/Δ^ alone compared to βCatCA-rescued mutant. Notably, this small set of 8 genes (Supplementary Data 1, yellow-shaded rows) included only three expected to act cell non-autonomously, *Dkk2*, *Notum* and *Sema3c* (Table 1, and boxed genes in yellow-shaded section of Supplementary Data set 1). *Sema3c* acts as a chemo-repulsive or chemo-attractive agent to guide neural cell migration^38^, especially neural crest, but not in digit patterning^39^. The Wnt pathway plays key roles in developing limb, including initiation/AER induction, digit patterning and tissue morphogenesis^4,10,19,40–43^. As expected, the 5’*Hoxd*^Δ/Δ^;βCatCA vs 5’*Hoxd*^Δ/Δ^ DE genes included multiple known canonical Wnt targets (Fig. 5d, f), such as *Sp5*, *Axin2*, and Wnt-antagonists involved in negative feedback (*Notum, Nkd1, Dkk2*). Of these, *Dkk2* and *Notum* act extracellularly. *Dkk2*, which is normally expressed in interdigits^41^, was both reduced in 5’*Hoxd*^Δ/Δ^ and highly upregulated by βCatCA rescue. *Notum*, which de-palmitoylates Wnt ligand extracellularly, was modestly upregulated in 5’*Hoxd*^Δ/Δ^ mutant and strongly upregulated by βCatCA rescue (Table 1).

To systematically consider any factor that could act cell non-autonomously from interdigits to restore interzone specification and joint formation, all DE genes in the 5’*Hoxd*^Δ/Δ^;βCatCA vs 5’*Hoxd*^Δ/Δ^ comparison alone were also filtered for extracellular location and function using GO^44^ and David (https://david.ncifcrf.gov/tools.jsp) tools, but excluding structural collagen genes. In addition to the three genes that were DE in both comparisons, we identified nine additional DE genes that act non-autonomously as secreted signaling factors, antagonists, or enzymes (Table 1 and boxed genes in green-shaded section of Supplementary Data set 1). Some of these were also DE in 5’*Hoxd*^Δ/Δ^ vs 5’*Hoxd*^*+*/Δ^ but did not reach statistical significance. Most notable in this group was the Bmp antagonist *Grem1*, induced 2.4-fold by βCatCA. Several other factors in this group are more involved in late morphogenesis (e.g., vascular, neural regulators). HCR fluorescent in situ validated differential expression (Supplementary Fig. 8) of the major candidate signaling factors identified (*Grem1, Dkk2, Notum*), as well as *Axin2*, a major canonical Wnt target induced by βCatCA, as predicted by DE analyses (Table 1, Fig. 5d). In summary, RNA profiling identified Wnt antagonists, *Dkk2* in particular, and possibly *Grem1* as candidate downstream factors for restoring interzone fate in βCatCA-rescued 5’*Hoxd*^Δ/Δ^ digits.

### Wnt antagonists restore interzone fate in 5’Hoxd^∆/∆^;βCatCA

Both Bmp and Wnt pathways have been implicated in regulating joint formation, and we assessed genetically whether the major candidates in either of these pathways identified by profiling played a causative role in restoring interzone fate in 5’*Hoxd*^Δ/Δ^ digits. The Bmp antagonist *Grem1* was increased by βCatCA (Table 1, Fig. 5d) and would be expected to inhibit activity of multiple Bmp ligands. We tested if raising the interdigit *Grem1* level could restore digit joint formation in 5’*Hoxd*^Δ/Δ^ digits by activating *RosaGrem1* with Hoxb6CreER^45^. In agreement with previous results^6^, although increasing *Grem1* expression alone in 5’*Hoxd*^Δ/Δ^ had a modest effect on phalanx length, it did not improve digit joint formation (*n* = 12/12, Supplementary Fig. 9).

**Fig. 6:**
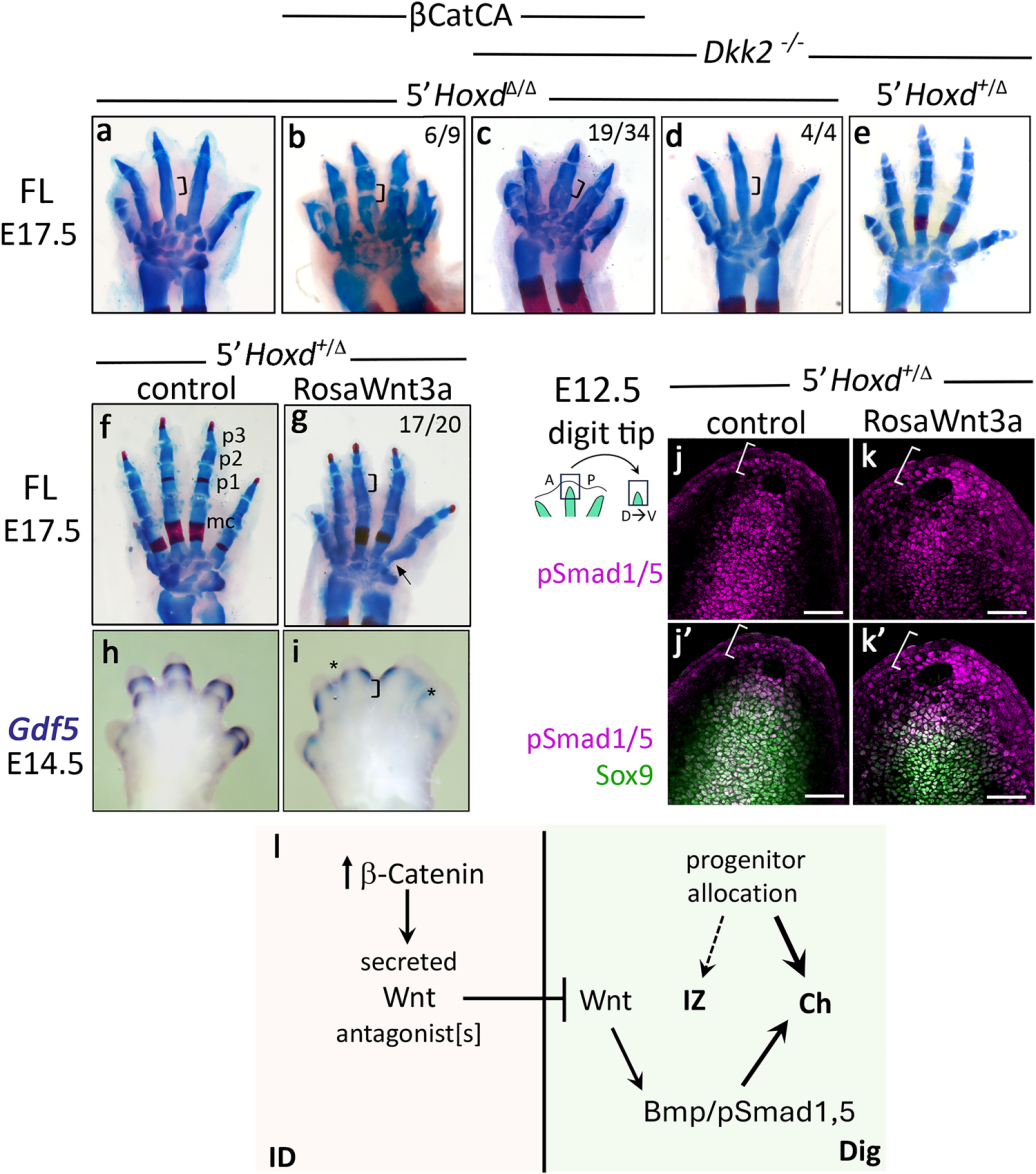
Effects of elevated Wnt signaling to digit tips on interzone and joint formation in 5’*Hoxd*^+/Δ^ control and in βCatCA-rescued 5’*Hoxd*^Δ/Δ^. **a–e** E17.5 skeletal stains showing that *Dkk2* deletion abolishes rescue of digit joints by interdigit βCatCA-activation in 5’*Hoxd*^Δ/Δ^ (compare (b) and (c)). βCatCA activated by Hoxb6CreER, 3 mg Tamoxifen injection at E11.25. Bracket indicates presumptive (a, c, d) or rescued (b) digit joint region. Numbers in panels indicate the frequency of phenotype shown in limbs analyzed for each genotype as indicated and collected from 9 independent litters. **f–i** Hoxb6CreER-activated RosaWnt3a transgene expression in 5’*Hoxd*^+/Δ^; 3 mg Tamoxifen injection at E10.75. (f, g) skeletal stain of E17.5 digits. Brackets indicate joint loss following Wnt3a transgene expression. Arrow indicates focal altered digit morphology. mc, metacarpal bone; p1-p3, phalanges 1–3. Numbers in panel (g) indicates the frequency of phenotype shown in total limbs analyzed and collected from 4 independent litters. (h, i) *Gdf5* in situ hybridization to visualize E14.5 interzones. Bracket shows loss of p1/p2 interzone. *indicates syndactyly. Three independent experiments analyzing *Gdf5*, each including 2 limbs per genotype and giving same results as shown. **j–k** pSmad1/5 immunostaining of E12.5 distal digit tip section (dorsoventral, as shown in schematic) of sibling controls and following Wnt3a-transgene activation. Brackets indicate sub-AER mesenchymal region. Two independent experiments analyzing pSmad1/5, each including 2 limbs per genotype and giving same results as shown. **j’, k’** merged images of pSmad1/5 and Sox9 immunofluorescence. Numbers in panels indicate the frequency of phenotype shown in the limbs analyzed. Scale Bar in ( j, j’, k, k’), 50um. **l** schematic summary of findings that canonical Wnt signaling to digit tips enhances pSmad1/5 activity, together with Bmps, and that Wnt antagonist (*Dkk2*) plays a role as downstream secreted factor in mediating interzone rescue by βCatCA.

Expression of canonical Wnt targets *Notum* and *Dkk2* were both increased by interdigital βCatCA and *Dkk2* was notably also reduced in 5’*Hoxd*^Δ/Δ^ interdigits. Both *Dkk2* and *Notum* knockout mice are viable and their limbs appear normal at birth^46,47^. However, multiple Wnt antagonists (including *Dkk2*) are expressed in interdigits and might act redundantly^41,48^. Since *Dkk2* is normally expressed selectively in interdigits and is both down-regulated in 5’*Hoxd*^Δ/Δ^ as well as up-regulated by βCatCA rescue (Table 1, Fig. 5d, Supplementary Fig. 8), we tested if *Dkk2* removal could block the joint restoration by interdigit βCatCA in 5’*Hoxd*^Δ/Δ^ digits. Deletion of *Dkk2* in sibling controls did not alter digit morphology including joint formation (Fig. 6e), and *Dkk2* deletion also did not further impair 5’*Hoxd*^Δ/Δ^ joint formation (*n* = 4/4, Fig. 6a, d), as expected since *Dkk2* expression is already barely detectable in 5’*Hoxd*^Δ/Δ^ interdigits compared to control. However, *Dkk2* gene deletion abolished the rescue of interzone formation by βCatCA in 5’*Hoxd*^Δ/Δ^;βCatCA embryos (*n* = 19/34, Fig. 6b,c-brackets), indicating that interdigit βCatCA rescues digit joint formation, at least in part, by restoring *Dkk2* expression in the 5’*Hoxd*^Δ/Δ^ interdigits. Consequently reducing, rather than activating, Wnt response in progenitor cells contributing to the nascent PFR plays a role in early interzone specification. Other negative feedback targets of the Wnt pathway, such as *Notum*, may also play a similar role.

To confirm this result, we checked whether elevating canonical Wnt ligand levels interferes with digit interzone specification in 5’*Hoxd*^*+*/Δ^ embryos, which would otherwise have a normal digit phenotype. Activating a conditional RosaWnt3a transgene using Hoxb6CreER, with early tamoxifen injection at E10.75 to achieve robust expression, caused frequent digit joint loss in 5’*Hoxd*^*+*/Δ^ embryos with reduced *Hoxd* gene dosage (*n* = 17/20, Fig. 6g), and rarely even in wild-type (5’*Hoxd*^*+/+*^) embryos (*n* = 1/10). In the case of Wnt ligand transgene activation, affected joints varied dependent on timing of tamoxifen treatment, with MCP more frequently compromised at earlier times and PIP affected with slightly later treatment (Fig. 6g). Enforced Wnt ligand expression also caused mild syndactyly and altered cartilage morphology (arrow,* in Fig. 6g, i), and *Gdf5* expression was absent in presumptive interzone regions at earlier stages (Fig. 6h, i brackets).

Together, the 5’*Hoxd* mutant (loss of Wnt antagonist) and transgenic Wnt gain-of-function results suggest that high canonical Wnt signaling to cells transiting into nascent PFR suppresses interzone specification, similarly to elevated Bmps. Indeed, immunodetectable pSmad1/5 was correspondingly elevated in the sub-AER mesenchyme and extending into the PFR region in Wnt3a transgenic embryos compared to sibling controls (Fig. 6j, k brackets). These results indicate that canonical Wnt signaling cooperates in some manner with the Bmp pathway to elevate pSmad1/5 activity in progenitor cells transiting to nascent PFR, thereby promoting chondrogenic- at the expense of interzone-specification, and suggest that interdigit Wnt antagonists promote interzone fate by inhibiting Wnt signaling to digit tips (Fig. 6l).

**Fig. 7:**
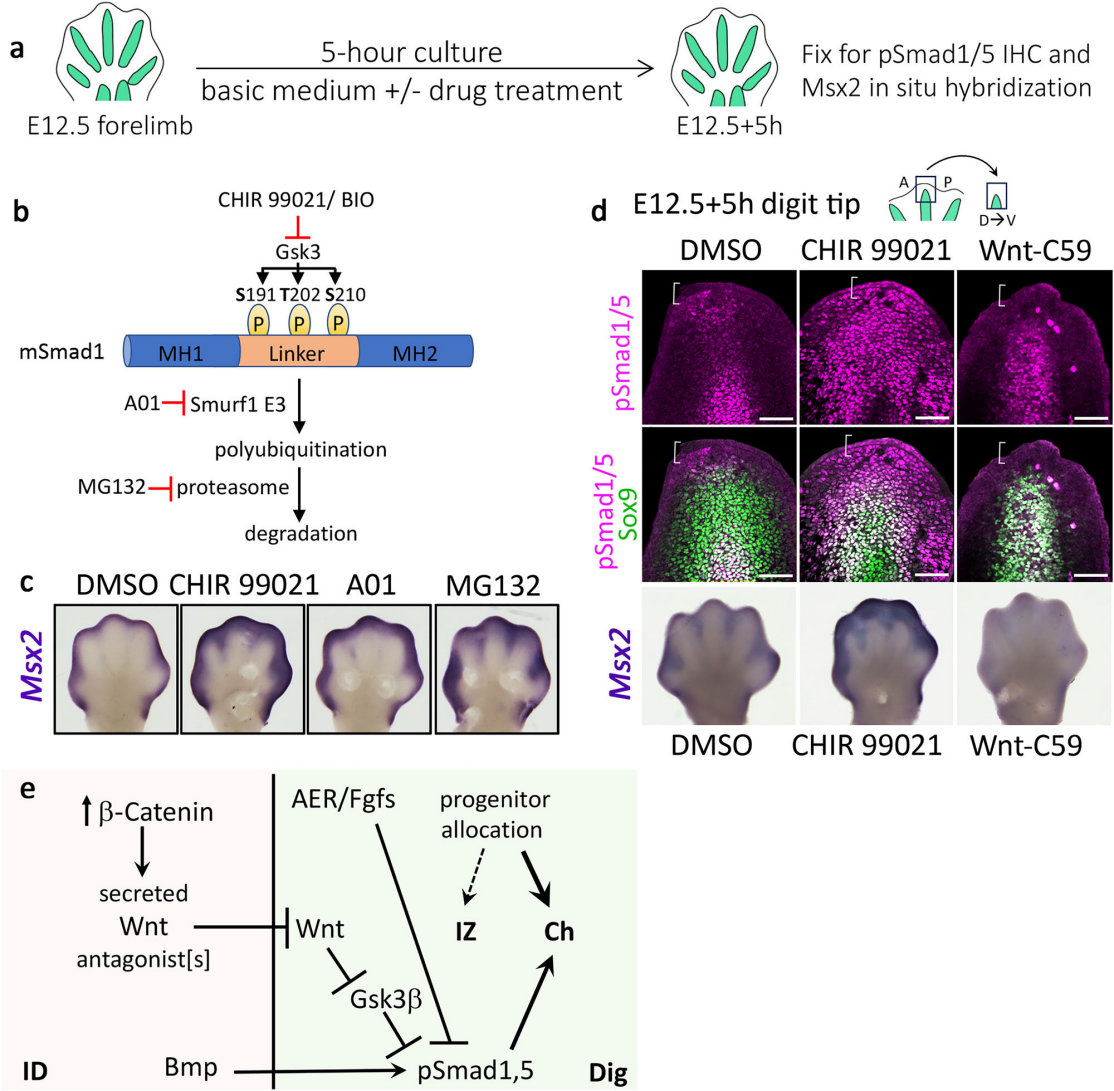
Wnt signaling enhances distal Bmp activity by inhibiting Gsk3β. **a** Experimental design of short-term E12.5 limb bud organ culture. **b** Schematic showing sites of action of different pharmacological agents used (adapted from ref. ^50^). **c** in situ hybridization of direct Bmp target *Msx2* shows enhanced Bmp activity after pharmacologic Gsk3 or proteasome inhibition, as indicated. Limb buds from four independent cultures, each including four limb buds treated with factors as indicated and analyzed for *Msx2*, gave the same results as shown. **d** Upper panels show pSmad1/5 and Sox9 immunostaining in digit tips at E12.5 + 5 h (dorsoventral section, as shown in schematic). Inhibiting Gsk3 activity increases pSmad1/5 detection in distal digit tip and conversely, inhibiting Wnt ligand secretion (Wnt-C59) reduces pSmad1/5 immunodetection. Limb buds from three independent cultures, each including three limb buds treated with factors as indicated and analyzed for pSmad1/5, gave the same results as shown. Bottom panel shows changes in Bmp response reported by expression of direct target *Msx2* RNA. Limb buds from 3 independent cultures, each including two limb buds treated with factors as indicated and analyzed for *Msx2*, gave the same results as shown. Scale Bar, 50 μm. **e** schematic summarizes findings that pharmacologic Gsk3 inhibition in cultured limb buds mimics effect of increased canonical Wnt activity in digit tip progenitors in vivo (see Fig. 6), enhancing pSmad1,5 detection in digit tips, and suggests that Wnts may cooperate with Bmps by inactivating Gsk3β, similar to the mechanism by which they activate/stabilize βCatenin (see text for details). We suggest that Gsk3β may act together with AER-Fgfs to destabilize pSmads since Gsk3β requires a phospho-peptide substrate and Fgf-activated MAPK can prime the Smad-linker region for Gsk3β action (see ref. ^50^).

The effect of excess canonical Wnt ligand in this context differs from that of direct βCatCA activation in digit tip, which acts mainly to block chondrogenesis during chondrogenic commitment (ref. ^6, 10^ and Fig. 1f, f’). Direct βCatCA activation (stabilized βCatenin)^23^ acts downstream of and bypasses the inhibition of the βCatenin destruction complex that occurs upon Wnt ligand-receptor binding. In the absence of Wnt ligand Gsk3β, a key component of this complex, phosphorylates the βCatenin nuclear transducer to target it for ubiquitination/degradation^49^. It has been shown that Gsk3β can also target receptor-activated pSmads for degradation by phosphorylating the pSmad linker region^50,51^ (see Fig. 7b) and consequently, Wnt ligand signaling to digit tip might stabilize and increase pSmad1/5 levels by inhibiting Gsk3β. Indeed, during mesoderm ventralization in gastrulating *Xenopus* embryos, canonical Wnt ligands were shown to act synergistically with Bmps by inactivating Gsk3β to stabilize pSmad1^50^.

### Inhibiting Gsk3β alone substitutes for Wnt signaling to enhance Bmp activity

We speculated that Wnt signaling also synergizes with Bmps to promote chondrogenic fate in digit tips by increasing receptor pSmad1/5 stability in progenitors transiting into nascent PFR, and consequently, that interdigit Wnt antagonists promote pSmad1/5 linker phosphorylation and turnover by stabilizing Gsk3β activity (Fig. 7e). To test this hypothesis, we used pharmacologic approaches in short-term whole limb bud organ culture at E12.5 (earliest digit-phalanx forming stage; Fig. 7a) to test if Gsk3β inhibition mimicked the effects of elevated Wnt ligand on pSmad1/5 levels in vivo. Bmp response level was assessed in short-term cultures of E12.5 limb buds both by pSmad1/5 protein (C-terminal activating phospho-serine) immunodetection and direct Bmp target *Msx2* RNA in situ hybridization. Short-term culture alone did not alter Bmp activity (*Msx2* expression comparable to freshly isolated contralateral limb buds, Supplementary Fig. 10).

To confirm that cultured limb buds were responsive to endogenous signals as in vivo, Wnt ligand activity was inhibited in culture by treatment with Wnt-C59, an inhibitor of porcupine that is essential for Wnt ligand secretion. Within several hours, Wnt-C59 exposure resulted in reduction of both immunodetectable pSmad1/5 protein and direct Bmp target *Msx2* RNA compared to controls (Fig. 7d), mimicking the effect of increased Wnt antagonists in vivo (Fig. 6b, c). Conversely, blocking Gsk3β activity with inhibitors BIO or CHIR effectively increased pSmad1/5 immunodetection and *Msx2* RNA levels (Fig. 7c, d, Supplementary Fig. 10). Furthermore, blockade of either the ubiquitin ligase Smurf1 (A01), or proteasome activities (MG132) (Fig. 7b, c) had very similar effects as Gsk3β inhibition, consistent with Gsk3β targeting pSmad1/5 for clearance by the same mechanism used for βCatenin in the destruction complex, and for Smad1 protein in *Xenopus*^50^. Together, these results suggest that Wnt signaling enhances Bmp activity in limb bud digit tips by inhibiting Gsk3β to stabilize pSmad1/5 proteins (Fig. 7e).

## Discussion

**Fig. 8:**
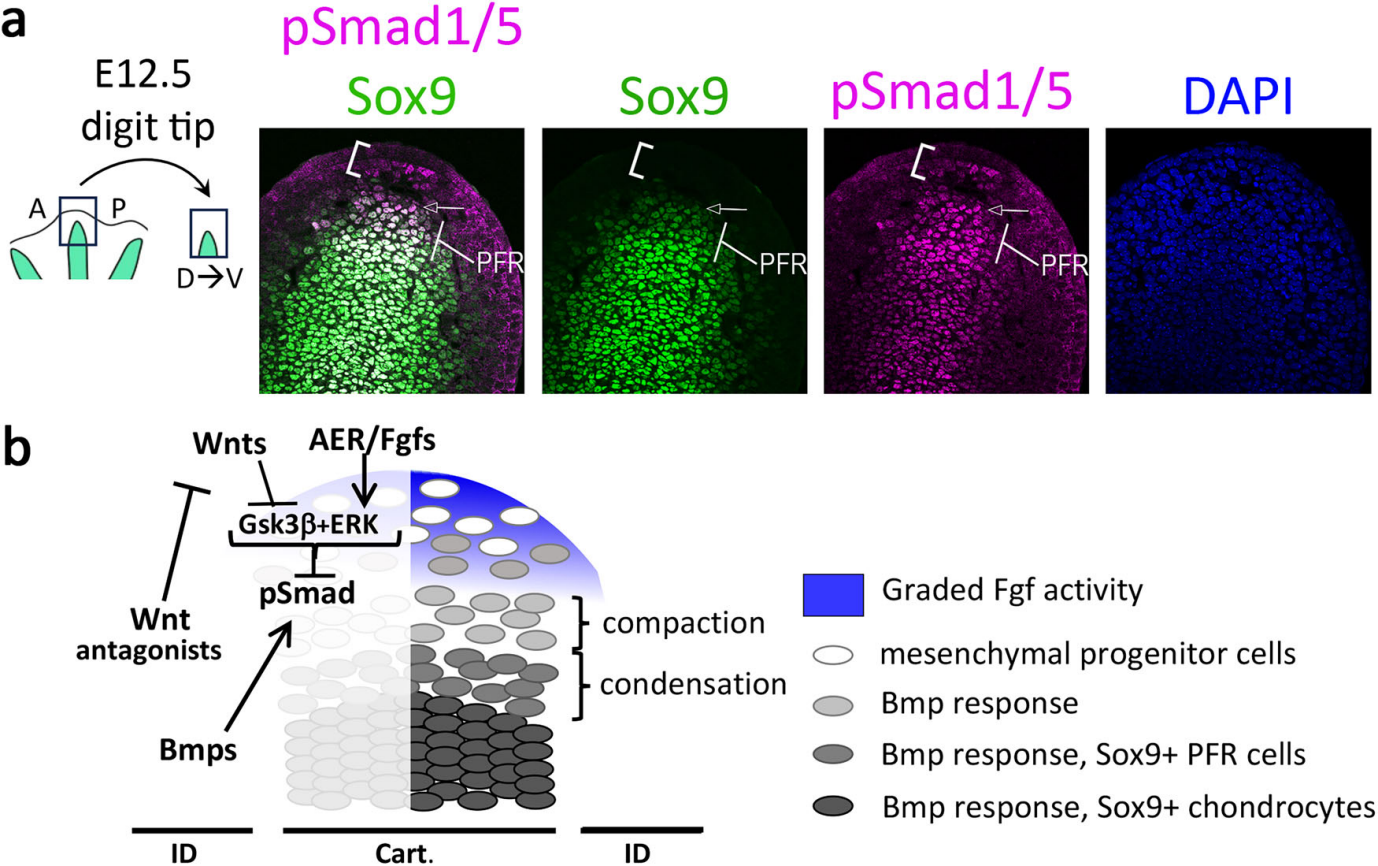
Bmp activation of pSmad1/5 precedes Sox9 expression as mesenchymal sub-AER progenitors transit to nascent PFR. **a** Sox9 and pSmad1/5 staining in wild-type E12.5 limb bud distal digit tip sections (dorsoventral, as shown in schematic). The bracket indicates sub-AER mesenchymal progenitors and the PFR zone is indicated below. Bmp activation of pSmad1/5 initiates in the sub-AER progenitors and promotes compaction of mesenchyme^11^ into nascent PFR (arrows), followed by coordinate phalanx (Sox9+)/interzone (Gdf5+) fate specification in PFR, dependent on Noggin activity level^6,15^. **b** Interaction of signaling pathways regulating the progression of mesenchymal progenitor cells to interzone or chondrogenic fate in nascent PFR. We propose that canonical Wnt activity stabilizes pSmad1/5 by suppressing downstream Gsk3β and thereby reducing Smad1/5 linker phosphorylation, whereas pERK activity downstream of AER/Fgfs acts in concert with Gsk3β to destabilize pSmad1/5^50^. Wnt antagonists counteract Wnt activity to prevent precocious and/or excess accumulation of pSmad1/5 in mesenchymal progenitors, which would impair the ability to reduce Bmp response and change fate during coordinate phalanx/interzone specification in established PFR (as occurs in *5’Hoxd*^Δ/Δ^ mutants or with RosaWnt3a transgene expression). See also Fig. 7 and discussion for details.

Previous work indicates that Wnts prevent mesenchymal cells from adopting a chondrogenic fate both by inducing silencing of the master regulator *Sox9*^9,10^ and subsequently by antagonizing Sox9 protein and directing its degradation^26,27^. Fgfs also repress *Sox9* but maintain the *Sox9* promoter in a poised state and prevent stable silencing^9^. In this study, we found instead that Wnts can augment Bmp activity causing mesenchymal digit tip cells to precociously adopt a pro-chondrogenic state favoring commitment to phalanx formation. Our RNAseq results and functional confirmation indicate that this unexpected Wnt-Bmp synergy is held in check by Wnt antagonists, such as Dkk2, which enable progenitors transiting to nascent PFR to remain developmentally plastic and adopt alternative chondrogenic or interzone fates. A proper balance between Wnt and Wnt-antagonist activities at this early phalanx initiation stage may maintain the *Sox9* locus silent but primed for expression, while also enabling early pSmad1/5 activation in response to Bmps to promote pre-chondrogenic mesenchymal cell compaction, a first step in PFR formation^11^ (Fig. 8a). Wnt antagonists would thus play key roles both in preventing precocious and/or excessive accumulation of pSmad1/5 by stabilizing Gsk3β in cells when Fgf levels are high, and in preventing irreversible *Sox9* locus silencing as Fgf levels decline by curtailing excess Wnt activity (Fig. 8b).

The two major 5’*Hoxd*^Δ/Δ^ digit phenotypes, shortening with phalanx loss (brachydactyly) and digit interzone loss, result from a perturbed balance between Wnt and Bmp pathways. Digit interzone specification is initiated in nascent PFR cells when the Bmp response is reduced^6^. Here we show that interdigit-activated βCatCA restores interzone fate non-autonomously in 5’*Hoxd*^Δ/Δ^ digits by inducing canonical Wnt targets that act as negative feedback regulators, particularly secreted Wnt antagonists including *Dkk2. Dkk2* is normally expressed in interdigits and is down-regulated in 5’*Hoxd*^Δ/Δ^ and up-regulated by rescue, and loss of *Dkk2* function abolishes the 5’*Hoxd*^Δ/Δ^ joint rescue by βCatCA. This indicates that digit interzone loss in the 5’*Hoxd*^Δ/Δ^ results, at least in part, from reduced Wnt antagonist levels and, consequently, elevated canonical Wnt activity. Indeed, transgenic *Wnt3a* ligand expression in 5’*Hoxd*^*+*/Δ^ digit tips during phalanx formation, like *Dkk2* loss in 5’*Hoxd*^Δ/Δ^;βCatCA, results in excess pSmad1/5 accumulation and digit interzone loss, suggesting that high Wnt activity impedes a switch to interzone fate. However, previous work on long bones has led to the proposal that canonical Wnt/βCatenin signaling induces interzone formation^4,19^. Overexpressed canonical Wnts induced ectopic *Gdf5+* interzone formation. However, *βCatenin* deletion or selective mesenchymal deletion of *c-Jun*, a transcriptional activator of Wnts in interzone^19,22,52^, did not abolish initial *Gdf5* expression in the emerging interzone of mutant mice. *Gdf5* expression only declined later, giving rise to secondary joint fusion phenotypes. The loss-of-function results indicate that interzones are still specified in these mutants and that canonical Wnt/βCatenin signaling is critical for subsequent interzone maintenance and maturation into joints, rather than initiation.

The presence of a transient circumferential ring of *Gdf5* expression in perichondrial tissue around phalangeal anlage (for MCP interzone) in 5’*Hoxd*^Δ/Δ^ digits, which is also seen in other mutants such as *Creb5*^31^ that affect interzone formation, raises the question of whether the phenotype reflects a joint maturation rather than early interzone specification defect. We feel that the early onset both of *Gdf5* appearance and sub-AER pSmad1/5 activity changes in 5’*Hoxd*^Δ/Δ^ digit tips indicates an early interzone induction defect. At present, it is unclear whether the circumferential expression is regulated separately and/or arises in a distinct *Gdf5*+ population. Whether it may provide additional spatial cues, perhaps for proper ligament positioning around joints, also remains to be determined.

Notably, our previous work indicated that reduction of phalanx number and interzone/joint loss phenotypes can be clearly uncoupled in different 5’*Hoxd*^Δ/Δ^ compound mutant contexts^6^. This is also evident in the results presented here. Indeed interdigit βCatCA restores only joint formation but not phalanx loss in 5’*Hoxd*^Δ/Δ^ digits. Detailed analysis of *Gdf5+* interzone appearance timing in the 5’*Hoxd*^Δ/Δ^ indicates that, unlike the proximal phalangeal MCP interzone, no residual middle phalangeal PIP *Gdf5*+ circumferential ring ever appears transiently, whereas the DIP interzone appears with the same timing in both the mutant and controls (Fig. 3, Supplementary Fig. 4). This is again consistent with the complete absence of a second (middle) phalanx which is not rescued by interdigit βCatCA. Noteably, a more drastic reduction of Bmp activity in the 5’*Hoxd*^Δ/Δ^ (reduced ligand availability and receptor dosage) can restore both triphalangy and PIP joints^6^. Further work will be required to determine what additional factors regulating digit mesenchymal progenitor maintenance or expansion may be altered following more extensive Bmp pathway down-modulation to restore phalanx formation in 5’*Hoxd*^Δ/Δ^ digits.

In the case of interzone and subsequent joint restoration by interdigit βCatCA, our results indicate that induced Wnt antagonists counteract the synergistic effect of canonical Wnts on pSmad1/5 activation by Bmps in sub-AER progenitors, resulting in biphalangeal digits with rescued joints. By inhibiting Gsk3β activity, elevated Wnt ligands prevent pSmad1/5 clearance, enhancing its accumulation and accelerating the pace of transition of mesenchymal progenitors to Sox9+ PFR cells and chondrogenic commitment. This impedes the switch to interzone fate as seen in 5’*Hoxd*^Δ/Δ^, or following Wnt3a misexpression, and leads to interzone loss. Conversely, reducing either Bmp activity^6^ or Wnt activity restores 5’*Hoxd*^*∆/∆*^ digit interzone specification.

Based on limb bud culture results with Gsk3β inhibitors, we propose a model in which pSmad1/5 stabilization occurs via canonical Wnt ligands acting to inhibit Gsk3β-mediated pSmad-linker phosphorylation. In principle, excess canonical Wnt signaling might act more indirectly, to reduce the level of noncanonical activity via competition for a shared effector^7,53^, since the noncanonical ligand Wnt5a plays a clear role in digit formation^53–56^. However, although phalanx formation is impaired in mutants with reduced Wnt5a activity, interzone formation is preserved^7,57^ and with complete loss of *Wnt5a* function, no PFR, and consequently no digit phalanx, forms at all^13,58^. Consequently, this mechanism seems unlikely to account for joint loss in the context of relative Wnt excess/Wnt antagonist reduction in 5’*Hoxd*^Δ/Δ^ digits. Both noncanonical Wnt receptor activity and Ihh signaling have also been implicated in promoting chondrogenic commitment in PFR, either by antagonizing the anti-chondrogenic effects of canonical Wnts on Sox9 or by inducing Bmps, respectively^7,59^. These activities promoting PFR phalanx commitment likely become important downstream of the Wnt antagonist role identified in this report, that maintains plasticity by constraining canonical Wnt activity levels in sub-AER progenitors transiting to nascent PFR, thereby preserving alternative paths to interzone or phalanx fate.

Wnts and Bmps have been proposed to play key roles in promoting digit regeneration^60,61^, and it is important to discern how blastema cells, the presumptive regenerative progenitor cells, integrate pathway activities to achieve full regeneration of both morphology and function. In this study we uncovered crosstalk between Bmps and Wnts and discovered that Wnt antagonists play a pivotal role in maintaining progenitor plasticity during the transition to PFR and phalanx formation in embryonic digits. These results shed light on how complex signaling interactions are balanced to regulate different specific digit identities (morphologies and phalanx/joint numbers) and help advance our understanding of how complex signaling interactions modulate cell fate determination during morphogenesis.

## Methods

### Mouse strains and embryo analyses

All animal studies were carried out according to the ethical guidelines of the Institutional Animal Care and Use Committee (IACUC) at NCI-Frederick and were approved under protocol #ASP-23-405. The *Catnb*^*+/Exon3Flox*^^23^ obtained from M. Taketo, *Hoxd*^*+/Del(11-13)*^(*5’Hoxd*^+/Δ^)^25^ obtained from D. Duboule, *NogginLacZ*^2^ obtained from R. Harland, *RosaGrem1*^45^ obtained from S. Vokes, Hoxb6CreER^28^ generated by S. Mackem, OsrCre^30^ obtained from G. Martin, *Dkk2*^*-/-*^(JAX #030130)^47^, *Sox9CreER*(*Sox9*^*CreER/+*^)^62^ obtained from H. Akiyama, *RosaWnt3a*^63^ obtained from T. Yamaguchi, and *Rosa-tdTomato*^64^ (JAX #007909) alleles used have all been reported previously. All mice were housed with a 12-h on/off light cycle (6 a.m.–6 p.m.), with temperature maintained at 68–79°F and humidity between 30% and 70%. For timed matings, noon on the day of post-coital plug was considered to be E0.5. For inducible Cre-drivers, a single dose of 3 mg tamoxifen was injected intraperitoneally^33^ at the time indicated in text, except in the case of lineage analysis. For *Sox9CreER* lineage analysis of *Sox9*+ digit tip cells (Fig. 2), the dosage was decreased to a single injection of 0.05 mg at the times indicated, to reduce the effective tamoxifen duration for recombination.

The 5’*Hoxd mutant (5’Hoxd*^Δ/Δ^) was maintained on an inbred FVB background, and digit phenotypes were very consistent. Interzone/joint formation was invariably lost in digits 3, 4 but was variably affected in digit 2 and digit 5 (~17% MCP loss in d2 and 32% loss in d5; as reported in ref. ^6^). In 5’*Hoxd*^*+*/Δ^ controls, d5 was rarely biphalangeal (approx. 5%) and no other abnormal digit phenotypes were observed. Our analyses focused on d3, d4 since these were most consistently abnormal in the 5’*Hoxd* mutant. Other mutant alleles used were commonly obtained on a mixed background and, to minimize the effects of background variation, all experiments comparing embryos having different mutant alleles were performed using sibling embryos obtained from the same litter. Embryos were not genotyped for gender; sufficient numbers were analyzed in experiments to be confident that both genders were represented.

### Skeletal preparation

Embryos were collected and fixed in ethanol, followed by acetone dehydration. The skeletons were stained in 0.3% Alcian Blue 8GS (Sigma-Alderich #A5268) and 0.1% Alizarin Red S (Sigma-Alderich #A5533) in 70% ethanol containing 5% acetic acid. Stained tissues were cleared in 2% potassium hydroxide and transferred to 50% glycerol for imaging^6^.

### in situ hybridization analysis of histologic sections

This procedure was carried out as described previously to visualize late skeletal-stage digit expression of *Gdf5*, *Wnt9a*, and joint maturation markers *Proteoglycan 4* (*Prg4*) and *Col IIA*^65^. Briefly, limbs were embedded in paraffin, and sectioned. Serial 5μm-thick sections were pretreated with 1 μg/ml proteinase K (Sigma-Aldrich #SAE0009) in 50 mM Tris-HCl, 5 mM EDTA pH 7.5 for 1 min at room temperature, immediately post-fixed in 4% paraformaldehyde buffer for 10 min, followed by washes and treatment with 0.25% acetic anhydride in triethanolamine buffer for 15 min. Sections were hybridized with antisense or sense [^35^S]-labeled riboprobes (approximately 1 × 10^6^ DPM/section) at 50 °C for 16 h. After hybridization, slides were treated with 20 μg/ml RNaseA for 30 min at 37 °C and dehydrated in ethanol, then coated with Kodak NTB-3 emulsion diluted 1:1 with water, air-dried and exposed in a light-proof box for image development. cDNA clones used for two isoforms of mouse Collagen Type II are: a 121 bp collagen IIA (284–404, NM_031163); a 356 bp collagen IIB (2409–2764, NM_031163)^66^.

### Whole mount colorimetric and Hybridization chain reaction (HCR) in situ analysis

Embryos were collected, fixed in 4% paraformaldehyde, and stored in 100% methanol until further analysis. The fixed embryos from desired genotypes were marked and combined in a single reaction tube to normalize conditions for subsequent colorimetric or HCR in situ analysis. The embryos were bleached in 5% hydrogen peroxide in methanol followed by rehydration and a brief proteinase K treatment (5–15 min, 20 μg/ml). For colorimetric in situ hybridization, embryos were hybridized with digoxigenin-UTP-labeled antisense riboprobes in a standard hybridization buffer containing 50% formamide, 1%SDS, 0.75 M NaCl at 70 °C overnight. Embryos were washed in hybridization buffer at 70 °C, hybridization buffer with reduced salt (0.15 M NaCl) at 50 °C, and in reduced salt (50 mM NaCl) without formamide at 70 °C, and then transferred to TBST (25 mM Tris pH 7.4, 150 mM NaCl, 0.1% Tween20) for incubation with anti-digoxigenin antibody conjugated to alkaline phosphatase (1:2000, Roche #11093274910) at 4 °C overnight. The colorimetric reaction was developed in BM Purple (Roche #11442074001) at room temperature until a clear signal was present. The whole mount HCR in situ hybridization followed the recommended procedures for third generation HCR probes^67^ designed by Molecular Instruments (Los Angeles, CA) and HCR fluorescence signals were analyzed by confocal microscopy.

### Immunofluorescence imaging

Embryos were collected, fixed in 4% paraformaldehyde and stored in 70% ethanol with phosphatase inhibitor cocktail (Millipore #524631) until use. Forelimb samples for tdTomato-labeled lineage tracing analysis were embedded in OCT, followed by cryo-sectioning at 10 μm thickness. Cryo-sections were stained with Alexa Fluor 488 conjugated anti-Sox9 antibody (1:500, Abcam #ab196450). For other immunofluorescent staining, fixed limb buds were embedded in 7% low-melting agarose for vibratome sections. For comparisons of signal intensity, the same master mix antibody solution was applied to each sample in an experimental set. 100 μm sections were treated with anti-phosphoSmad1/5 (1:200, Cell Signaling #9516) and visualized with Alexa Fluor 594 secondary antibody, or stained with Alexa Fluor 488 conjugated anti-phosphoSmad3 (1:300, Abcam #ab310954), followed by counterstaining with either Alexa Fluor 488 conjugated anti-Sox9 antibody or with Alexa Fluor 647 conjugated anti-Sox9 antibody (1:500, Abcam #ab196184), respectively, to visualize chondrogenic condensations. Stained samples were imaged by confocal microscopy. At least 3 independent samples of each genotype were imaged for analyses.

### Short-term limb bud culture with pharmacological agents

Embryonic stage E12.5 mouse forelimb buds were dissected in ice-cold PBS, followed by incubation in serum-free limb bud minimal media^68^ either with DMSO vehicle alone (0.01%, Sigma-Aldrich #8418), CHIR 99021 (10 μM, Tocris #4423), BIO (5uM, Tocris #3194), A01 (5 μM, MedChemExpress #HY-110195), MG132 (50 μM, Tocris #1748) or Wnt-C59 (10 μM, Tocris #5148) at 37 °C with 5% CO_2_ for 5 h. After 5 h, cultured limb buds were fixed in 4% paraformaldehyde and stored in 100% methanol for subsequent analysis by immunofluorescence staining of vibratome sections or colorimetric whole mount in situ analysis as described above. At least 5 independent limb buds were treated and analyzed for each condition.

### Bulk RNAseq analysis

To generate different genotypes for transcriptome comparison, Hoxb6CreER^tg/tg^;5’*Hoxd*^*+*/Δ^ males were crossed with *Catnb*^+*/Exon3Flox*^;5’*Hoxd*^Δ/Δ^;*Rosa26*^tdTomato/tdTomato^ females. Pregnant females were injected with Tamoxifen at E11.25 and embryos were collected at early E12.5 and held at 4 °C in PBS during rapid PCR genotyping. For each genotype of sibling embryos (5’*Hoxd*^*+*/Δ^, 5’*Hoxd*^Δ/Δ^ or 5’*Hoxd*^Δ/Δ^;βCatCA), forelimb buds of at least three to four embryos from two litters were grouped. Interdigit tissues (between d2-d3, d3-d4, and d4-d5, see Fig. 5a) were dissected with tungsten needles and dissociated with 0.25% Trypsin (Gibco #15090046). After 3–4 min incubation at 37 °C followed by gentle trituration, trypsin was inactivated with 1 mM AEBSF (MP Biomedicals #193503) followed by addition of 1% serum and isolation of tdTomato-labeled interdigit cells by FACS. Three independent experimental sets were collected and processed for bulk RNAseq (see also Supplementary Information). Barcoded libraries prepared from cDNAs of nine samples were pooled and sequenced for paired-end 103 bp reads using Illumina TruSeq. Reads were trimmed of adaptors and low quality bases using Trimmomatic software (v0.35) and aligned to the mm9 release of the mouse genome (NCBI137, July 2007) using TopHat2 (v2.1.0/v2.1.1). Differential expression (DE) analysis was carried out using DESeq2^69^ with default parameters including two-sided hypothesis test and normalized expression counts were generated using the median of ratios method (Bioconductor 3.1/3.2, DESeq2 version 1.8-1.10). Normalized counts for complete data in Supplementary data set 2 was used for PCA visualization; for all other analyses Supplementary data set 1 was used (significant DEGs from Suppl. data set 2 with FC ≥ 1.5; FDR ≤ 0.1). 3D-PCA was performed using plotly.js. version 2.0, 2D-PCA and volcano plots using ggplot2 version 4.0.3, and heatmaps using pheatmap version 1.0.13. The DAVID Functional Annotation Tool (https://david.ncifcrf.gov/tools.jsp) was used to identify secreted/extracellular factors in the DE gene sets as potential candidates involved in joint rescue. Gene Ontology for biological process enrichments analysis was carried out with clusterProfiler R package version 4.10.0^44^ and results adjusted for significance based on Benjamini-Hochberg with a Q value cutoff of 0.01, and otherwise using default parameters.

### Reporting summary

Further information on research design is available in the Nature Portfolio Reporting Summary linked to this article.

## Supplementary information

Supplementary Information

Peer Review file

Description of Additional Supplementary Files

Supplementary Data 1

Supplementary Data 2

Reporting Summary

## Data Availability

All raw sequence data and aligned reads from bulk RNAseq experiments analyzed in this study are freely available from the GEO repository under accession number GSE317957, with no restrictions. The normalized RNAseq counts for each gene for each of the three independent biological replicates of three genotypes analyzed (5’*Hoxd*^+/Δ^, 5’*Hoxd*^Δ/Δ^, and 5’*Hoxd*^Δ/Δ^;βCatCA) as well as the entire DEseq2 analysis summarized visually in Fig. 5, and Supplementary Figs. 6 and 7 are included in the Supplementary Data sets 1 and 2.
