# Supplementary material for: A pivotal Wnt antagonist role promoting digit joint specification by constraining Wnt activity
Source: Nat Commun. 2026 May 26;17:6835. doi: 10.1038/s41467-026-73549-4 (PMC13389162; doi:10.1038/s41467-026-73549-4)
Supplement: Supplementary file 1 — Supplementary Information [file 41467_2026_73549_MOESM1_ESM.pdf]

Supporting Information List:

Supplementary information on annotation of biological replicates included in RNAseq data (GSE 317957) and in Suppl. data sets 1, 2, and in visualized expression data of each replicate presented in Fig. 5b, and Suppl. Figs. 6, 7.

Supplementary Figure 1. Time course of Tamoxifen treatment for Hoxb6CreER-activated interdigit βCatCA to effectively restore 5'Hoxd$^{\Delta/\Delta}$ digit joint formation.

Supplementary Figure 2. Lineage analysis shows that βCatCA-expressing cells do not contribute significantly to cartilage or joint region after Hoxb6CreER activation, and are absent from rescued digit 3 joints in OsrCre-activated βCatCA.

Supplementary Figure 3. βCatCA expression in interdigits restores joint formation in 5'Hoxd$^{\Delta/\Delta}$ digits.

Supplementary Figure 4. Detailed time course of sequential Gdf5+ interzone appearance during phalanx formation in 5'Hoxd$^{+/\Delta}$(control), 5'Hoxd$^{\Delta/\Delta}$, and 5'Hoxd$^{\Delta/\Delta}$;βCatCA digit tips from E12.5 - E14.5.

Supplementary Figure 5. ActivinβA expression and Tgfβ activity (pSmad3) appear unchanged between 5'Hoxd$^{+/\Delta}$, 5'Hoxd$^{\Delta/\Delta}$ and 5'Hoxd$^{\Delta/\Delta}$;βCatCA in distal digit tip region.

Supplementary Figure 6. 2D principle component (PC) analysis comparing PC1-3 contributions shows distinct segregation of 3 biological replicates for each genotype indicated (color-coded).

Supplementary Figure 7. Heatmaps of gene expression levels in each of 3 biological replicates for DE genes in pairwise comparisons of three genotypes indicated.

Supplementary Figure 8. HCR in situ validation of differentially expressed RNAs for Wnt- and Bmp-pathway secreted factor candidates identified in transcriptome analysis.

Supplementary Figure 9. Transgenic CreER-activated Grem1 misexpression does not restore 5'Hoxd$^{\Delta/\Delta}$ digit joint formation.

Supplementary Figure 10. Bmp activity levels (Msx2 RNA) following 5hrs of short-term limb bud organ culture.

Supplementary Information on annotation of biological replicates of 3 genotypes compared in RNAseq analysis.

For labeled samples in PCA plots (Figure 5b and Supplemental Figure 6) and heatmaps (Supplemental Figure 7) which show visual representations of RNAseq expression data for individual replicates (not averages of 3 replicates), the short-hand annotation uses the same numbering order as the data sets of complete and highlighted DEseq2 analysis for the 3 genotypes compared, and in the primary raw sequence data deposited in the GEO repository (#GSE317957).

Sample sets 1, 2.1 (or 2.2), and 3 are independent experiments in which embryos of 3 different genotypes (indicated below) were collected and processed together (see Methods for details), with each genotype included in pooled sibling embryos obtained from several litters (5-6 embryos per genotype included). Biological replicate #2 is annotated as 2.1 for pooled 5'Hoxd$^{+/\Delta}$ and 5'Hoxd$^{\Delta/\Delta}$ embryo tissues and annotated as 2.2 for βCatCA-rescued mutant tissues because the cDNA library had to be re-generated from the cDNA from that replicate owing to a technical error by the NCI Genomics Core, and was sequenced separately (this replicate is designated as 2* in the relevant figures).

**genotype:**	**shorthand names in DEseq2 datasets 1,2:**	**designation in PCAs, heatmaps:**
5'Hoxd$^{+/\Delta}$	Del_Het (1, 2.1, 3)	1, 2, 3
5'Hoxd$^{\Delta/\Delta}$	Del_Hom (1, 2.1, 3)	1, 2, 3
5'Hoxd$^{\Delta/\Delta}$;βCatCA	Del_hom_Exlox (1, 2.2, 3)	1, 2*, 3

Supplementary Figure 1

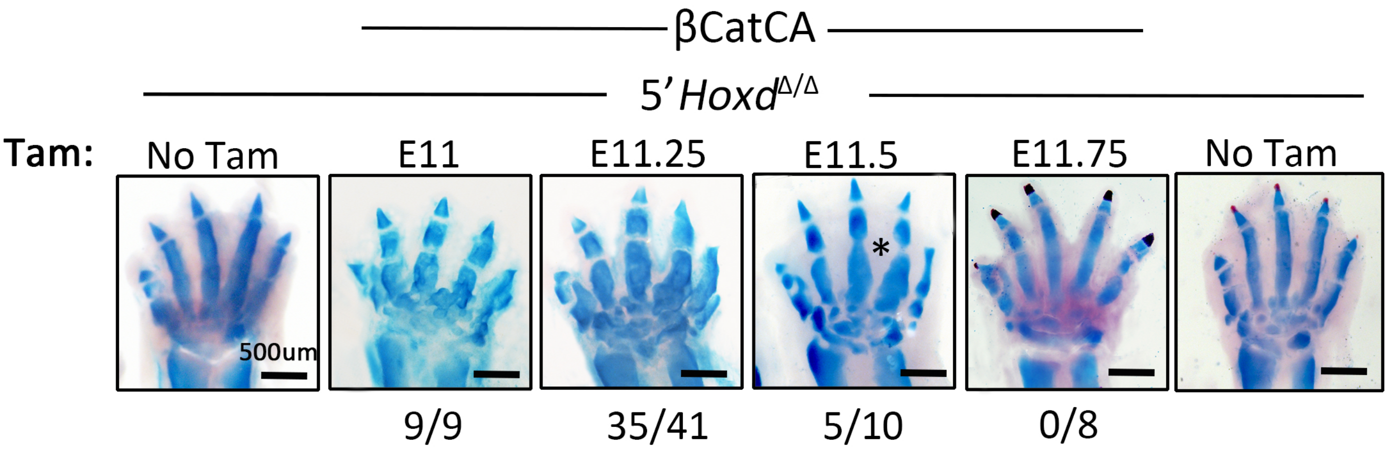

Supplementary Fig. 1. Time course of Tamoxifen treatment for Hoxb6CreER-activated interdigit βCatCA to effectively restore 5'*Hoxd*$^{\Delta/\Delta}$ digit joint formation.

Tamoxifen (Tam, 3mg dose per pregnant dam) injection times indicated above for images of forelimb skeletons from embryos collected at either E16.5 (or at E17.5 for two far-right panels only). Efficacy of joint restoration is gradually reduced with treatment ranging from E11 to 11.5 and is completely lost by E11.75. Numbers below each image indicate limbs with completely or partially (*) restored metacarpo-phalangeal joints/total limbs analyzed for that tamoxifen treatment time. For each treatment time, from E11-E11.75, embryos were collected from 1, 6, 4, and 3 independent litters respectively. Scale Bar, 500um

Supplementary Figure 2

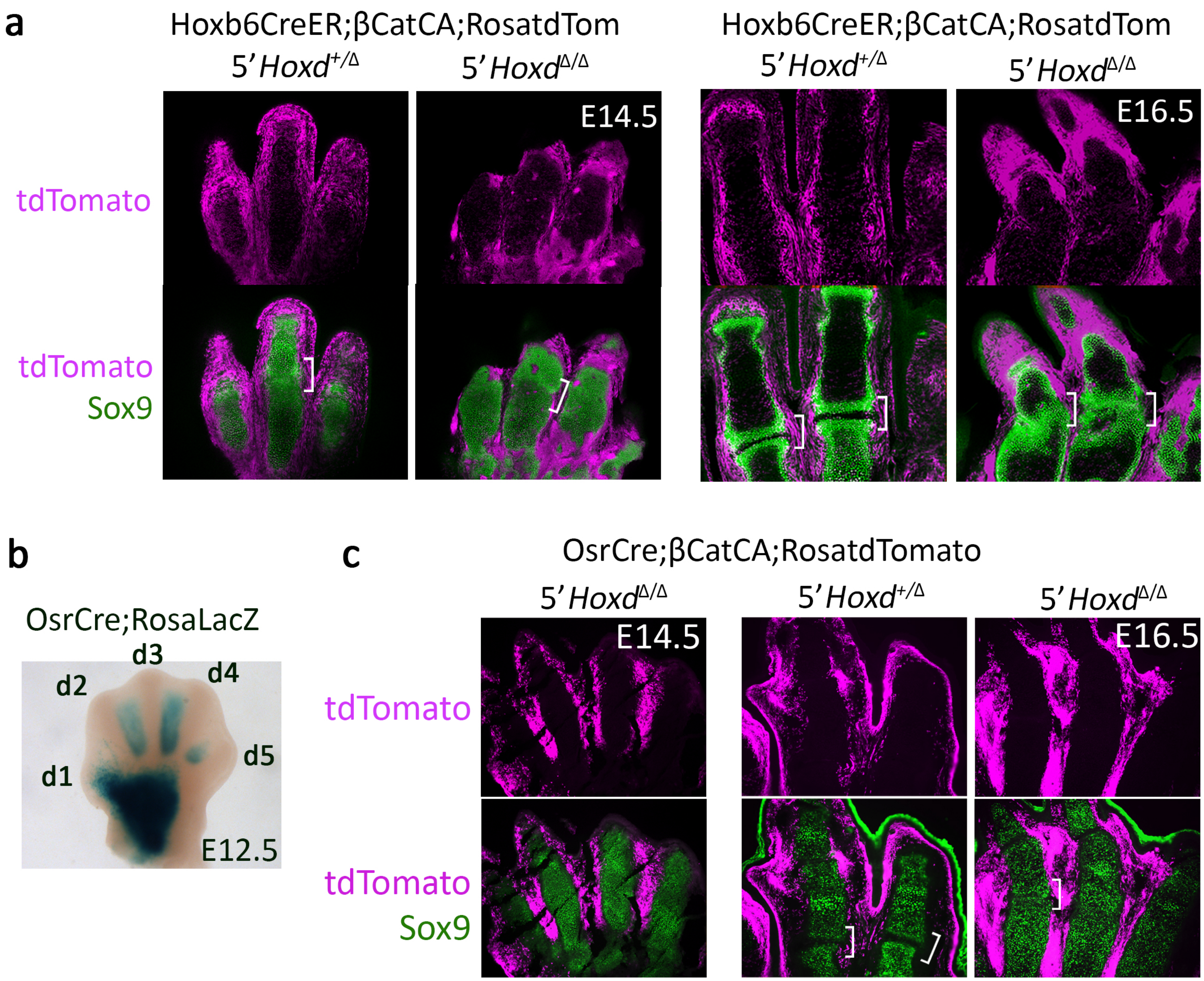

Supplementary Fig. 2. Lineage analysis shows that βCatCA-expressing cells do not contribute significantly to cartilage or joint region after Hoxb6CreER activation, and are absent from rescued digit 3 joints in OsrCre-activated βCatCA.

a Hoxb6CreER;Rosa-tdTomato labeled (pseudocolored magenta) cells in 5′*Hoxd*$^{\Delta/\Delta}$;βCatCA embryos are present predominantly in interdigit tissues with rare Tomato-expressing cells in cartilage observed at E14.5 or E16.5, after tamoxifen treatment at E11.25 (3mg dose per pregnant dam). Embryos were collected from one litter for each stage and 2 embryo limbs examined for each genotype indicated, with same results as shown. Images are optical sections from 200um vibratome section immunostained for Sox9 protein. Brackets indicate metacarpo-phalangeal joints. **b** OsrCre;RosaLacZ activity is strong in interdigits flanking digit 3 (d3) metacarpal condensation by E12.5 in forelimb, but very weak in other interdigits at this stage. **c** OsrCre;Rosa-tdTomato labeled (pseudocolored magenta) cells in 5′*Hoxd*$^{\Delta/\Delta}$;βCatCA embryos are observed only in interdigit tissues at E14.5 and E16.5. Embryos collected from 2 independent litters at E14.5 with 2 embryos limbs examined for each genotype and embryos collected from one litter at E16.5 with 2 embryos limbs examined for each genotype, all with same results as shown. Images from 5um cryo-sections immunostained for Sox9 protein. Brackets indicate metacarpo-phalangeal joints. Note lack of contribution of OsrCre-activated βCatCA expressing cells to rescued interzone/joint.

Supplementary Figure 3

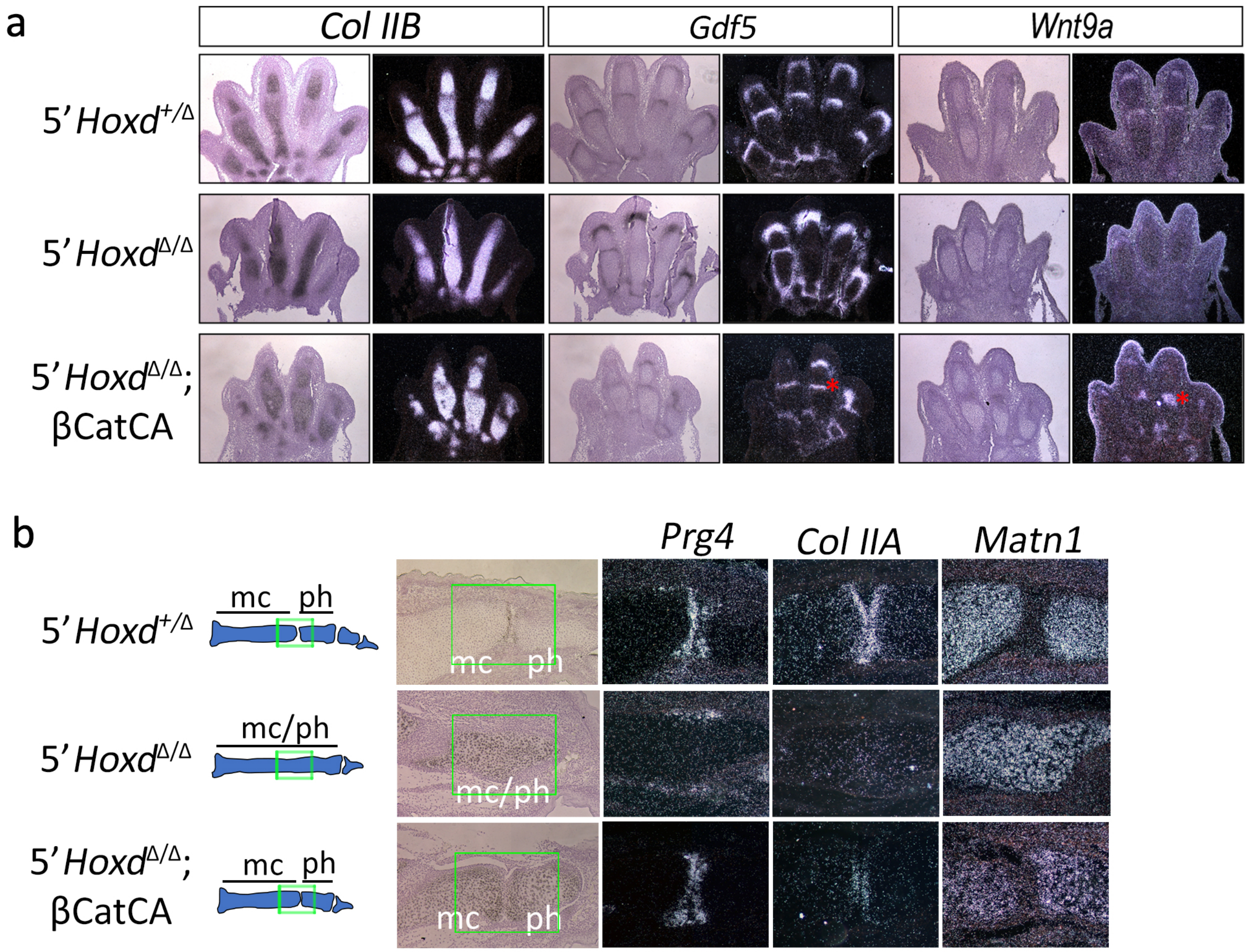

Supplementary Fig. 3. βCatCA expression in interdigits restores joint formation in 5'*Hoxd*$^{\Delta/\Delta}$ digits.
a In 5'*Hoxd*$^{\Delta/\Delta}$ E14.5 digits, interzone expression of *Gdf5* and *Wnt9a* are absent, but are restored by activation of βCatCA in the interdigits with Hoxb6CreER (3mg tamoxifen injected at E11.25). *Collagen type IIB (Col IIB)* is expressed in condensed cartilage[65,66]. *, restored interzone. **b** In 5'*Hoxd*$^{\Delta/\Delta}$ P0 (newborn) digits, *Proteoglycan 4 (Prg4)* is only expressed laterally at the periphery of the presumptive joint region, but is restored within the articular cartilage by OsrCre-activated βCatCA in interdigits, indicating normal joint maturation. *Col IIA*, which detects Exon 2 residues, is expressed in articular cartilage[65,66] and is absent in 5'*Hoxd*$^{\Delta/\Delta}$ digits but also restored by βCatCA activation. *Matrilin 1* (*Matn1*) is expressed in cartilage matrix. ph: phalanx; mc: metacarpal bone. Paraffin sections from 6-8 embedded limbs of each genotype indicated at E14.5 (a) and at P0 (b) were mounted and hybridized with probe indicated (2-4 slides with 4-8 sections/slide for each probe), and each experiment performed in triplicate gave same results as shown.

Supplementary Figure 4

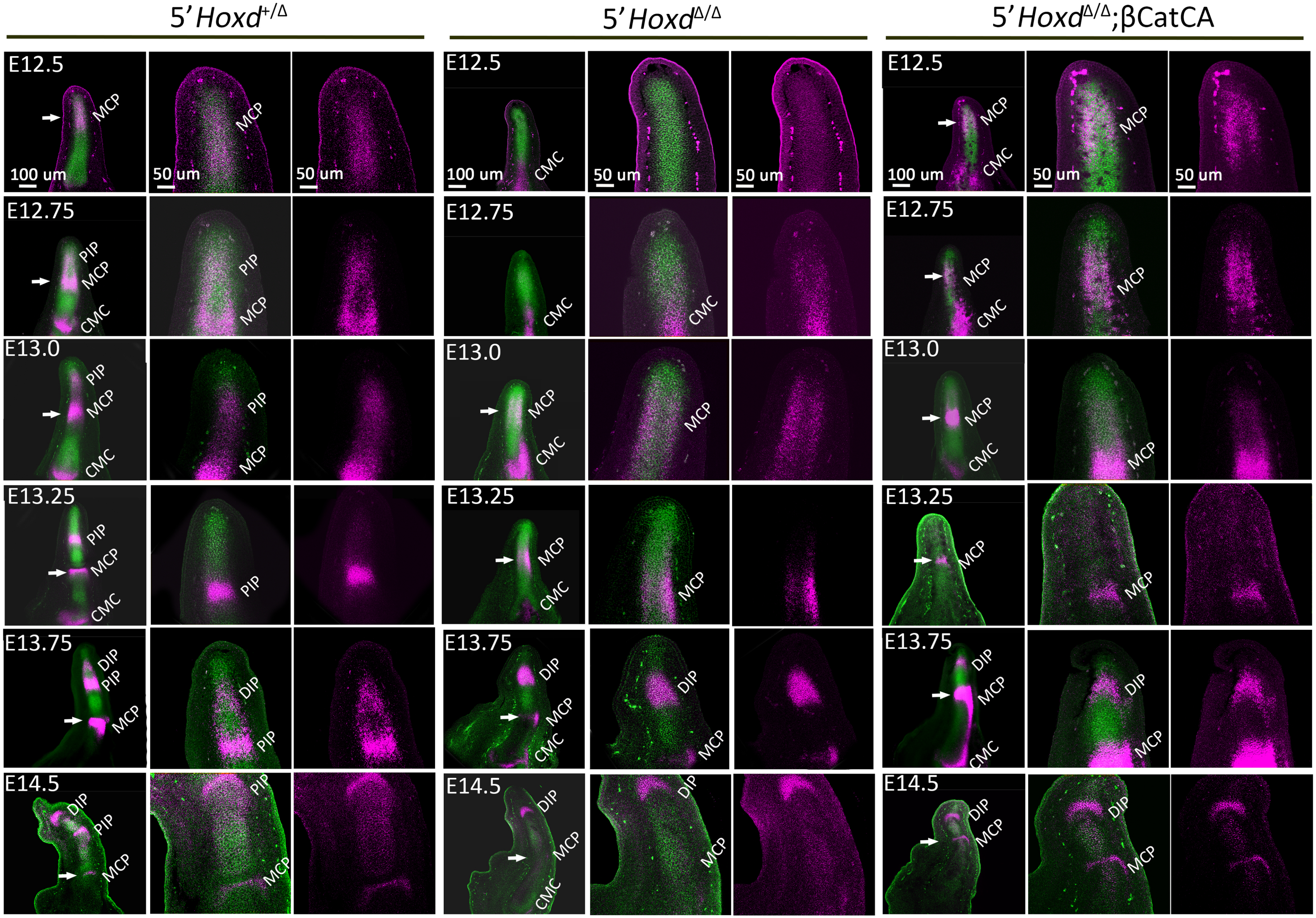

Supplementary Fig. 4. Detailed time course of sequential *Gdf5*+ interzone appearance during phalanx formation in 5′*Hoxd*+/Δ(control), 5′*Hoxd*Δ/Δ, and 5′*Hoxd*Δ/Δ;βCatCA digit tips from E12.5 - E14.5. Additional nonmerged, higher magnification (mag) images for data summarized in Figure 3. HCR i*n situ* staining of merged *Gdf5* (pseudocolored magenta) and *Sox9* (green) at low mag including entire metacarpal region to digit tip or distal metacarpal end to digit tip (for some E13.75-14.5 panels), and higher mag digit tip region images of merged and *Gdf5* (magenta) only channels from E12.5-E14.5. Arrows indicate MCP interzone position. CMC, carpometacarpal; MCP, metacarpophalangeal; PIP, proximal interphalangeal; DIP, distal interphalangeal interzones. Scale bars of 100um (shown in E12.5 panels) apply to all lower mag images, and scale bars of 50um apply to all higher mag images.

Supplementary Figure 5

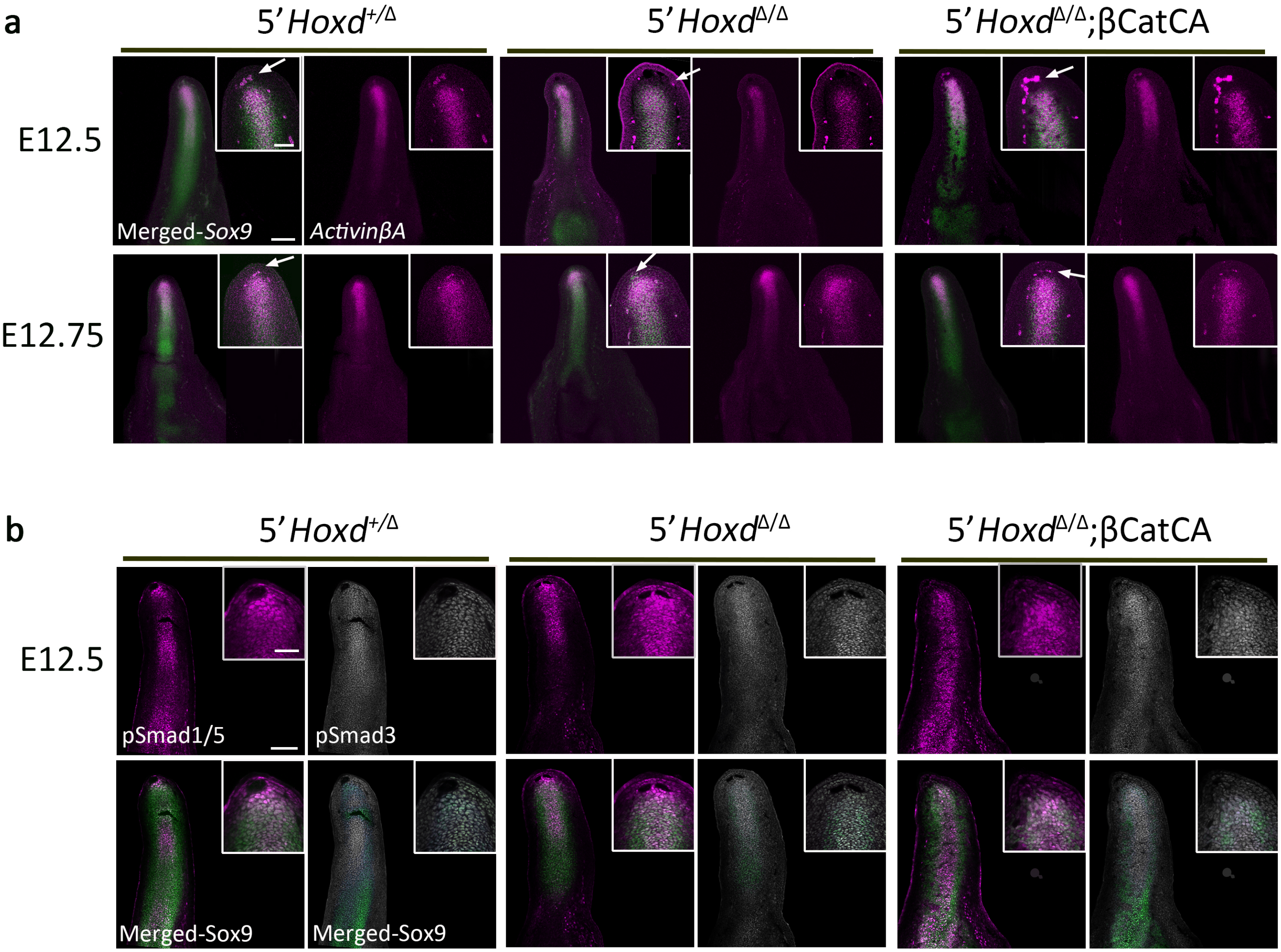

Supplementary Fig. 5. *ActivinβA* expression and Tgfβ activity (pSmad3) appear unchanged between 5′*Hoxd* $^{+/\Delta}$, 5′*Hoxd* $^{\Delta/\Delta}$ and 5′*Hoxd* $^{\Delta/\Delta}$;βCatCA in distal digit tip region.

a HCR *in situ* RNA staining of *ActivinβA* (pseudocolored magenta) and *Sox9* (green) expression in digit tips at E12.5 and E12.75 for three different genotypes shown. Scale bars shown in E12.5 *ActivinβA*/*Sox9* merged control panel represent 100um for low magnification image or 50um for higher magnification inset and apply to all other panels or insets. Arrows indicate erythrocyte autofluorescence. **b** Immunodetection of pSmad1/5 (pseudocolored magenta) or pSmad3 (pseudocolored white) and Sox9 (green, in lower merged image panels) in digit tips at E12.5 for three genotypes as shown. Scale bars shown in pSmad1/5 control panel represent 100um for low magnification image or 50um for higher magnification inset and apply to all other panels or insets. For all experiments, Hoxb6CreER-activated βCatCA was generated by Tamoxifen treatment (3mg dose) at E11.25. E12.5 embryos for both (a) and (b) collected from 3 litters analyzed in 3 independent experiments including 2 embryos for each genotype indicated per experiment. E12.75 embryos for (a) collected from 2 litters analyzed in 2 independent experiments including 2 embryos for each genotype indicated per experiment.

Supplementary Figure 6

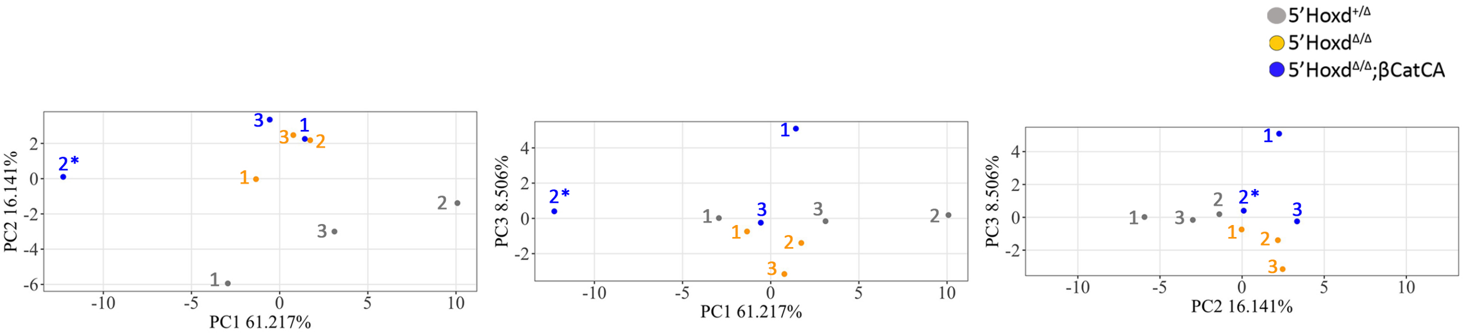

Supplementary Fig. 6. 2D principle component (PC) analysis comparing PC1-3 contributions shows distinct segregation of 3 biological replicates for each genotype indicated (color-coded). Note that plot of PC2 vs PC3 indicates PC3 contributes to distinction between 5'*Hoxd* mutant and βCatCA-rescued mutant. See Supplementary information for detailed annotation of replicates 1-3 and explanation of outlier 2* replicate. Normalized counts for complete transcriptome data of replicates 1-3 in Supplementary data set 2 were generated using median of ratios method (Bioconductor 3.1/3.2, DESeq2 version 1.8-1.10) and PCA plots performed using plotly.js. version 2.0 (3-D, Fig. 5a), and ggplot2 version 4.0.3 (2-D).

Supplementary Figure 7

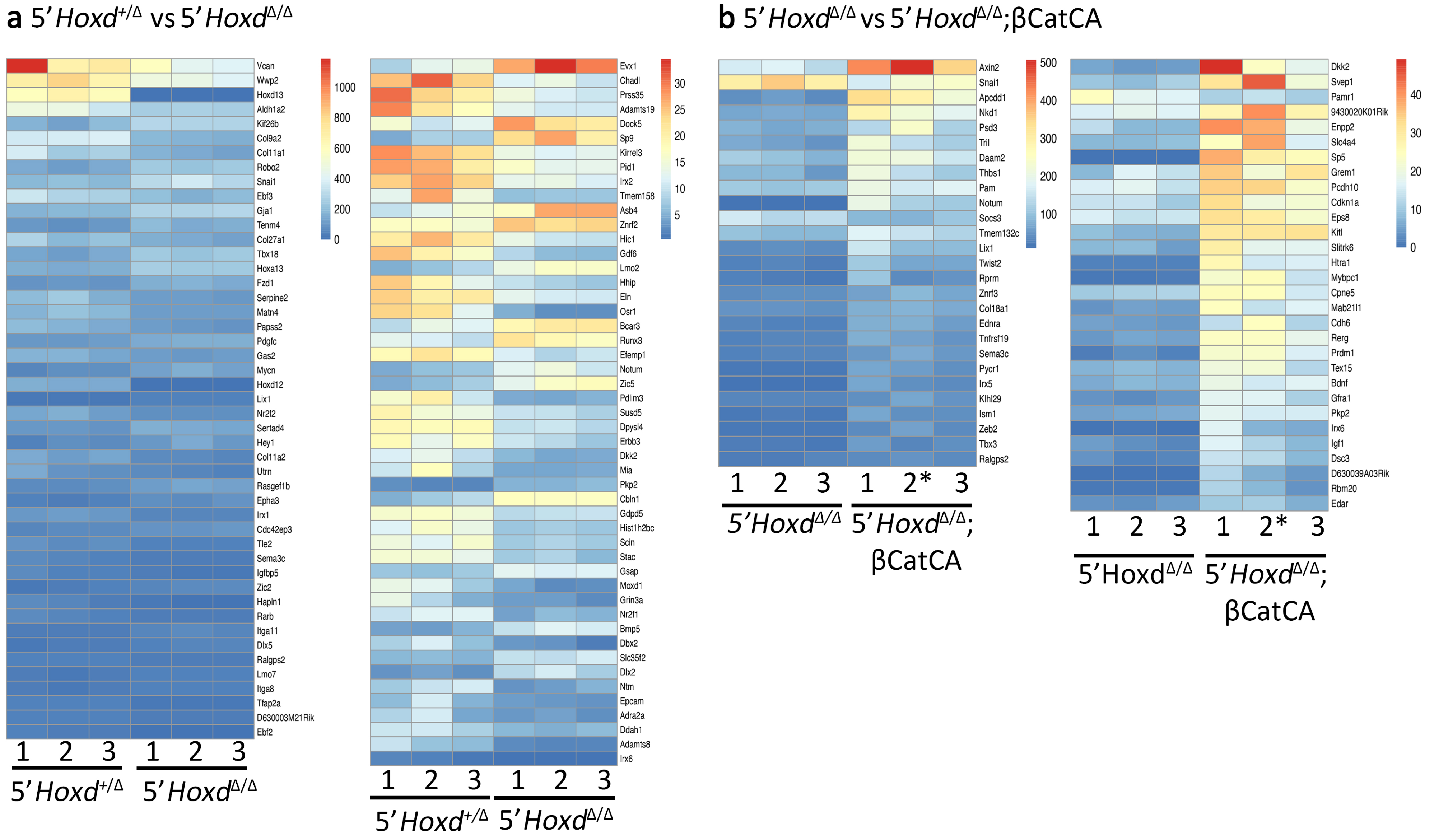

Supplementary Fig. 7. Heatmaps of gene expression levels in each of 3 biological replicates for DE genes in pairwise comparisons of three genotypes indicated. Heatmaps generated using pheatmap version 1.0.13 from normalized expression data of replicates 1-3 of each genotype for DEGs in Supplementary data set 1 and summarized in Fig. 5. Owing to the wide range of expression levels for different genes, heatmaps are split into two groups so gene expression differences are more easily appreciated visually. See Supplementary information for detailed annotation of replicates 1-3 and explanation of outlier 2* replicate. Normalized counts generated using median of ratios method (Bioconductor 3.1/3.2, DESeq2 ver. 1.8-1.10).
a 5'*Hoxd*$^{+/\Delta}$ vs 5'*Hoxd*$^{\Delta/\Delta}$; heatmaps split according to genes with average expression greater than, or less than 38.
b 5'*Hoxd*$^{\Delta/\Delta}$ vs 5'*Hoxd*$^{\Delta/\Delta}$;βCatCA ; heatmaps split according to genes with average expression greater than, or less than 49.

Supplementary Figure 8

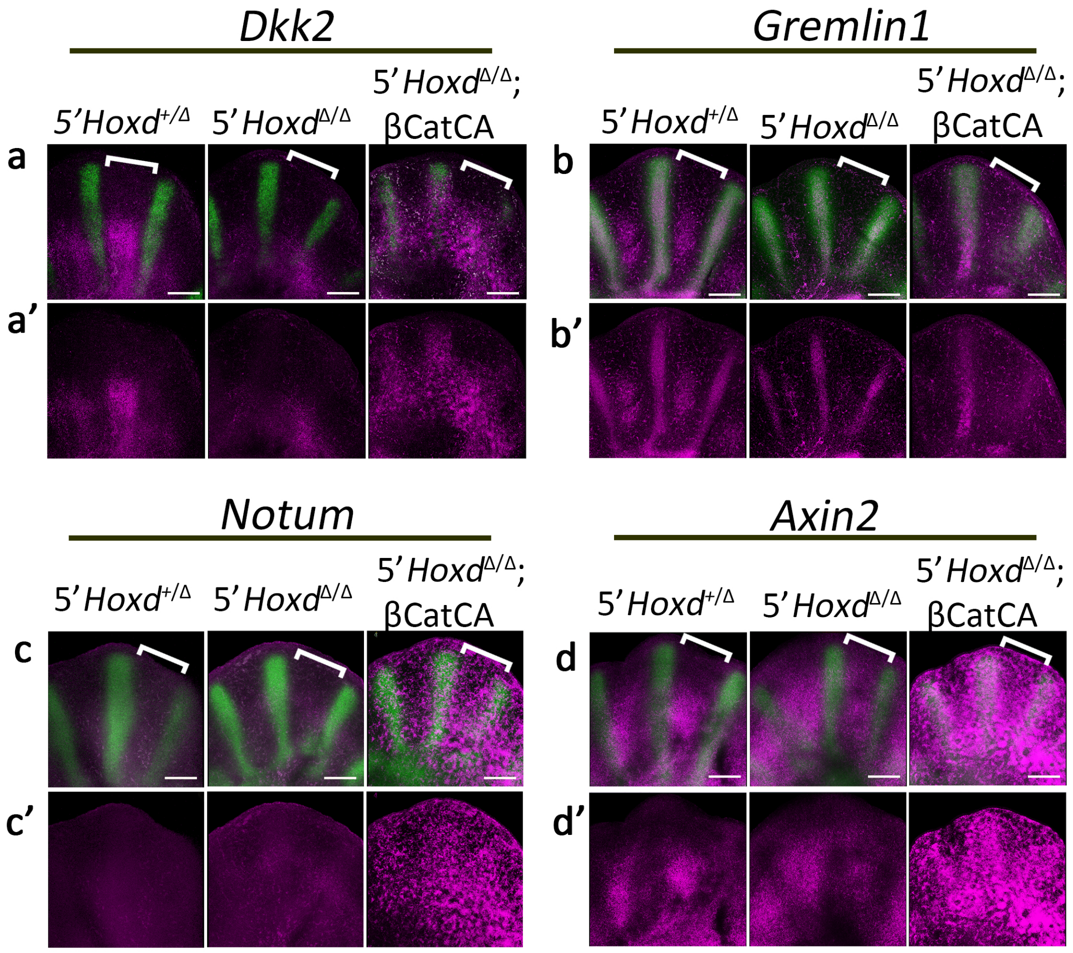

Supplementary Fig. 8. HCR i*n situ* validation of differentially expressed RNAs for Wnt- and Bmp-pathway secreted factor candidates identified in transcriptome analysis.
a-d merged images of fluorescent HCR *in situ* staining for *Sox9* (in green) and for differentially expressed genes (pseudocolored magenta) as indicated, in E12.5 forelimb buds. **a'-d'** fluorescent HCR *in situ* staining for differentially expressed genes alone. Brackets indicate interdigit tissue between digit 3-4 to highlight gene expression changes seen between control (5′$Hoxd^{+/\Delta}$), 5′$Hoxd^{\Delta/\Delta}$ and 5′$Hoxd^{\Delta/\Delta}$ rescued by Hoxb6CreER-activated βCatCA (3mg tamoxifen at E11.25). Two independent experiments analyzing expression of each target gene as indicated included 2 limb buds for each genotype indicated per experiment and gave the same results as shown.
Scale bar, 200um for all panels.

Supplementary Figure 9

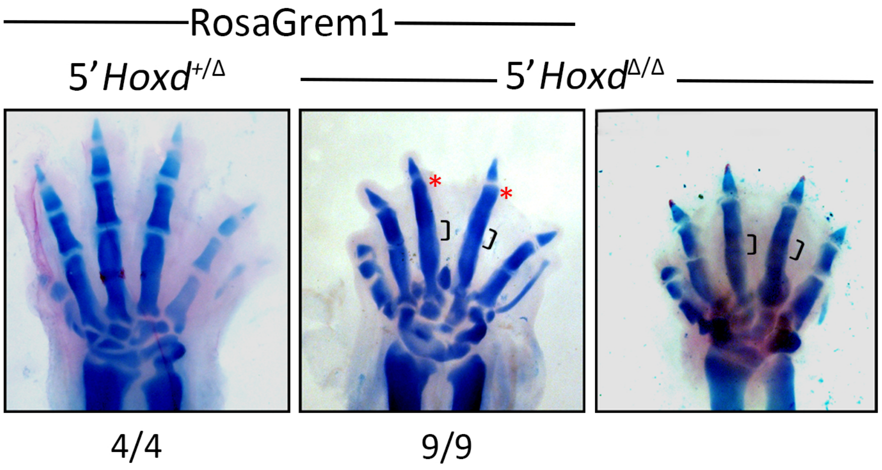

Supplementary Fig. 9. Transgenic CreER-activated Grem1 misexpression does not restore 5′*Hoxd*$^{\Delta/\Delta}$ digit joint formation. No significant effect of RosaGrem1 activation by Hoxb6CreER (3mg tamoxifen at E10.75) were observed on 5′*Hoxd* $^{\Delta/\Delta}$ digit joint restoration or causing digit morphological alterations in 5′*Hoxd* $^{+/\Delta}$ siblings. Image of 5′*Hoxd* $^{\Delta/\Delta}$ (no Grem1 transgene), which has completely penetrant (100%) loss of MCP joints in digits 3,4 in multiple strain backgrounds, shown for comparison. Numbers below RosaGrem1+ images indicate the number of limbs out of total analyzed with the phenotype shown for that genotype, in embryos collected from 4 independent litters. Skeletal stain of E16.5 forelimbs shown. *indicates modest effect on digit length. Brackets indicate presumptive MCP joint regions that fail to form.

Supplementary Figure 10

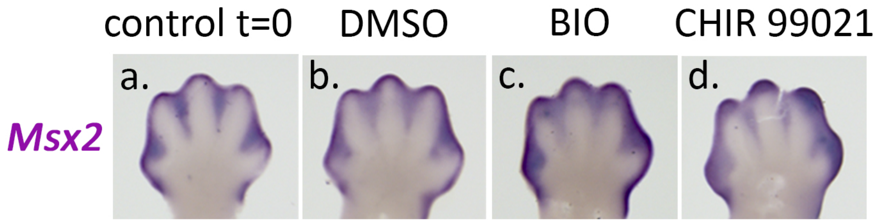

Supplementary Fig. 10. Bmp activity levels (*Msx2* RNA) following 5hrs of short-term limb bud organ culture.

a freshly isolated E12.5 limb bud prior to culture. **b** E12.5 limb bud cultured for 5hrs with vehicle alone (DMSO). **c,d** E12.5 limb bud cultured for 5hrs with Gsk3 inhibitor BIO (c) or CHIR99021 (d). Bmp activity (reported by direct Bmp target *Msx2* RNA) is not substantially altered during short-term limb bud organ culture (compare panel (b) with (a)), but is augmented by inhibition of Gsk3 activity during culture (compare (b) with (c), (d)). Two independent experiments analyzing Msx2 RNA in limb buds, treated for 5hrs in culture as indicated, included 2 limb buds for each treatment per experiment and gave the same results as shown.
