# Supplementary material for: A pivotal Wnt antagonist role promoting digit joint specification by constraining Wnt activity
Source: Nat Commun. 2026 May 26;17:6835. doi: 10.1038/s41467-026-73549-4 (PMC13389162; doi:10.1038/s41467-026-73549-4)
Supplement: Supplementary file 3 — Description of Additional Supplementary Files [file 41467_2026_73549_MOESM3_ESM.pdf]

Description of Additional Supplementary Files

File Name: Supplementary Data 1
Description: DE analysis for all genes in Figure 5C Venn diagram.

File Name: Supplementary Data 2
Description: Complete normalized replicate counts and DE analysis.
