# Supplementary material for: A pivotal Wnt antagonist role promoting digit joint specification by constraining Wnt activity
Source: Nat Commun. 2026 May 26;17:6835. doi: 10.1038/s41467-026-73549-4 (PMC13389162; doi:10.1038/s41467-026-73549-4)
Supplement: Supplementary file 6 — Reporting Summary [file 41467_2026_73549_MOESM6_ESM.pdf]

Corresponding author(s): Susan Mackem

Last updated by author(s): May 5,2026

Reporting Summary

Nature Portfolio wishes to improve the reproducibility of the work that we publish. This form provides structure for consistency and transparency in reporting. For further information on Nature Portfolio policies, see our Editorial Policies and the Editorial Policy Checklist.

Statistics

For all statistical analyses, confirm that the following items are present in the figure legend, table legend, main text, or Methods section.

n/a	Confirmed	
☐	☒	The exact sample size (*n*) for each experimental group/condition, given as a discrete number and unit of measurement
☒	☐	A statement on whether measurements were taken from distinct samples or whether the same sample was measured repeatedly
☐	☒	The statistical test(s) used AND whether they are one- or two-sided<br>*Only common tests should be described solely by name; describe more complex techniques in the Methods section.*
☒	☐	A description of all covariates tested
☒	☐	A description of any assumptions or corrections, such as tests of normality and adjustment for multiple comparisons
☐	☒	A full description of the statistical parameters including central tendency (e.g. means) or other basic estimates (e.g. regression coefficient) AND variation (e.g. standard deviation) or associated estimates of uncertainty (e.g. confidence intervals)
☐	☒	For null hypothesis testing, the test statistic (e.g. *F*, *t*, *r*) with confidence intervals, effect sizes, degrees of freedom and *P* value noted<br>*Give P values as exact values whenever suitable.*
☒	☐	For Bayesian analysis, information on the choice of priors and Markov chain Monte Carlo settings
☒	☐	For hierarchical and complex designs, identification of the appropriate level for tests and full reporting of outcomes
☒	☐	Estimates of effect sizes (e.g. Cohen's *d*, Pearson's *r*), indicating how they were calculated

Our web collection on statistics for biologists contains articles on many of the points above.

Software and code

Policy information about availability of computer code

Data collection	Three independent experimental sets were collected and processed for bulk RNAseq. Barcoded libraries prepared from 9 samples of cDNAs were pooled and sequenced for paired-end 103bp reads on Illumina TruSeq. No specific software used in data collection.
Data analysis	Reads for each sample were aligned to the mm9 release of mouse genome (NCBI137) using TopHat2 (v2.1.0/v2.1.1).<br>Differential Expression (DE) analysis carried out using DESeq2 (Bioconductor 3.1/3.2, DESeq2 version 1.8-1.10) with default parameters and normalized counts generated using the median of ratios method.<br>3D-PCA generated using plotly.js. version 2.0,<br>2D-PCA and volcano plots generated using ggplot2 version 4.0.3.<br>Heatmaps generated using pheatmap version 1.0.13.<br>Gene Ontology for biological process enrichments analysis performed with clusterProfiler R package version 4.10.044 and adjusted for significance based on Benjamini-Hochberg with a Q value cutoff of 0.01, and otherwise using default parameters.

For manuscripts utilizing custom algorithms or software that are central to the research but not yet described in published literature, software must be made available to editors and reviewers. We strongly encourage code deposition in a community repository (e.g. GitHub). See the Nature Portfolio guidelines for submitting code & software for further information.

Data

Policy information about availability of data

All manuscripts must include a data availability statement. This statement should provide the following information, where applicable:

- Accession codes, unique identifiers, or web links for publicly available datasets
- A description of any restrictions on data availability
- For clinical datasets or third party data, please ensure that the statement adheres to our policy

All raw sequence data and aligned reads from bulk RNAseq experiments analyzed in this study are freely available from the GEO repository (GSE317957) https://www.ncbi.nlm.nih.gov/geo/query/acc.cgi?acc=GSE317957, with no restrictions. The normalized RNAseq counts for each gene for each of the three independent biological replicates of three genotypes analyzed, as well as the entire DEseq2 analysis summarized visually in Figure 5, and Supplementary Figures 6, 7 are included in the Supplementary Data sets 1 and 2.

Research involving human participants, their data, or biological material

Policy information about studies with human participants or human data. See also policy information about sex, gender (identity/presentation), and sexual orientation and race, ethnicity and racism.

Reporting on sex and gender	NA
Reporting on race, ethnicity, or other socially relevant groupings	NA
Population characteristics	NA
Recruitment	NA
Ethics oversight	NA

Note that full information on the approval of the study protocol must also be provided in the manuscript.

Field-specific reporting

Please select the one below that is the best fit for your research. If you are not sure, read the appropriate sections before making your selection.

☒ Life sciences ☐ Behavioural & social sciences ☐ Ecological, evolutionary & environmental sciences

For a reference copy of the document with all sections, see nature.com/documents/nr-reporting-summary-flat.pdf

Life sciences study design

All studies must disclose on these points even when the disclosure is negative.

Sample size	For mutant skeletal phenotype assessments, multiple independent samples/embryos were analyzed from multiple litters (indicated in legends) and numbers reported in each figure. Results were highly comparable for the same genotypes in independent litters. No statistical analyses were performed. For molecular analyses, at least 2-3 independent experiments including at least 2 or more samples of each genotype were performed, that again all yielded comparable results. For bulk RNAseq, 3 independent biological replicates were included, following ENCODE recommended guidelines of at least 2 replicates (https://www.encodeproject.org/data-standards/encode4-bulk-rna/#standards).
Data exclusions	No data was excluded from analysis.
Replication	For each mutant tissue sample (limb bud cells) analyzed, at least 4, but usually more than 10 independent samples of each genotype were examined for gross skeletal morphology, and at least 5 samples for molecular (in situ RNA, protein) analyses. The results for a given genotype were detailed in figures and legends and were always very comparable. All results were reproduced in multiple experiments with similar results.
Randomization	Comparisons of mutant and controls cannot be randomized since knowledge of the genotypes is required to ensure appropriate numbers of each are included in an experiment and embryos of different genotypes are marked in a simple manner (+tail bud, no tail bud) so that all genotypes with multiple embryos could be analyzed under identical experimental conditions (eg. hybridization or immunostaining together in a single reaction chamber) or pooling of several samples of the same genotype to generate RNA for one RNAseq analysis replicate. The experiments in this study compare complex mutants and control sibling embryos receiving the same treatment (eg. transgene addition), so randomization according to the treatment given is not relevant to the study. Controlling for background strain variation was achieved by comparing sibling embryos with different genotypes from the same litters, to maximize strain similarity.
Blinding	Blinding is not possible at the time of collection because low frequency, complex genotypes must be determined immediately to ensure adequate representation of all relevant genotypes (mutant, control, each with and without a new genetic test modification introduced) in each experiment. Likewise, to ensure uniformity of genetic background and other variables (for eg. age of collection, timing of tamoxifen treatment, RNA probe or antibody incubation conditions), mutant and control comparisons were performed between sibling embryos with different genotypes processed together. This would restrict blinding to evaluating a very large number of paired samples, each having a very

small total sample number (often only 3-4) to compare, and would be of very limited value.
For genome-wide analyses such as RNAseq, blinding is not required (see ENCODE guideline link above).

Reporting for specific materials, systems and methods

We require information from authors about some types of materials, experimental systems and methods used in many studies. Here, indicate whether each material, system or method listed is relevant to your study. If you are not sure if a list item applies to your research, read the appropriate section before selecting a response.

Materials & experimental systems

n/a	Involved in the study	
☐	☒	Antibodies
☒	☐	Eukaryotic cell lines
☒	☐	Palaeontology and archaeology
☐	☒	Animals and other organisms
☒	☐	Clinical data
☒	☐	Dual use research of concern
☒	☐	Plants

Methods

n/a	Involved in the study	
☒	☐	ChIP-seq
☒	☐	Flow cytometry
☒	☐	MRI-based neuroimaging

Antibodies

Antibodies used: anti-Sox9 antibody (1:500 dilution, Abcam, catolog#: ab196450); anti-phosphoSmad1/5 (1:200 dilution, Cell Signaling, catalog#: 9516) ; anti-phosphoSmad3 (1:300 dilution, Abcam, catalog#: ab310954)

Validation: anti-Sox9 Manufacturer statement and link: Alexa Fluor® 488 Anti-SOX9 antibody [EPR14335] (ab196450) is a rabbit recombinant monoclonal antibody and is validated for use in Flow Cytometry (Intra), Flow Cytometry (Flow Cyt), Immunocytochemistry/ immunofluorescence (ICC/IF) in Human, Mouse samples.
https://www.abcam.com/en-us/products/primary-antibodies/alexa-fluor-488-sox9-antibody-epr14335-ab196450; anti-pSmad1/5 Manufacturer statement and link: Phospho-SMAD1/5 (Ser463/465) (41D10) Rabbit Monoclonal Antibody detects endogenous levels of SMAD1 and SMAD5 only when dually phosphorylated at Ser463 and Ser465 and is also predicted to detect SMAD9 (SMAD8) when phosphorylated at Ser465 and Ser467. This antibody does not cross-react with other SMAD-related proteins. Species Reactivity: Human, Mouse, Rat; https://www.cellsignal.com/products/primary-antibodies/phospho-smad1-5-ser463-465-41d10-rabbit-monoclonal-antibody/9516?srsltid=AfmBOooPw98sylCwpuy6NApzcciWWTcKUW7uwHaw7u4oTbhStK4zr_dH ; anti-pSmad3 Manufacturer statement and link: Anti-SMAD3 (pS423/425) detects Smad3 phosphorylated on Serine 423 and Serine 425 and was developed by Abcam using patented rabbit monoclonal antibody technology and is validated for use in ChIC/CUT&RUN-seq, Dot Blot, ICC/IF, IHC-P and WB. https://www.abcam.com/en-us/products/primary-antibodies/smad3-ps423-425-smad5-ps463-465-smad1-ps463-465-smad2-ps465-467-antibody-ep823y-ab52903 .

Animals and other research organisms

Policy information about studies involving animals; ARRIVE guidelines recommended for reporting animal research, and Sex and Gender in Research

Laboratory animals: Catnb+/Exon3Flox obtained from M. Taketo, Hoxd+/Del(11-13)(5'Hoxd+/) obtained from D. Duboule, NogginLacZ obtained from R. Harland, RosaGrem1 obtained from S. Vokes, Hoxb6CreER generated by S. Mackem, OsrCre obtained from G. Martin, Dkk2-/-(JAX #030130), Sox9CreER(Sox9CreER/+) obtained from H. Akiyama, RosaWnt3a obtained from T. Yamaguchi, and Rosa-tdTomato (from JAX #007909). The sources we obtained these from are the investigators that generated these lines (or from JAX as indicated). References for the creation and characterization of each of these lines are included in the text Methods. The background strains for these lines are mixed. To maximize similarity of control and mutant background, sibling mutant and control embryos from the same litter were compared for phenotypic and gene expression differences.
All experiments used embryos that were collected at specific post-coital ages indicated in the text, figure panels, and figure legends. Females used in crosses to generate embryos were between 6-10 weeks in age.

Wild animals: None used.

Reporting on sex: Embryos were not genotyped to determine gender and no exclusions were made based on gender. Generally, sufficient embryos were analyzed to make it likely that both genders were represented (usually at least 10 per genotype for skeletal phenotypes).

Field-collected samples: None used.

Ethics oversight: All experiments and procedures carried out were approved by NIH IACUC at NCI-Frederick under protocol #ASP-23-405.

Note that full information on the approval of the study protocol must also be provided in the manuscript.

Plants

Seed stocks	NA
Novel plant genotypes	NA
Authentication	NA
