# Supplementary material for: A pivotal Wnt antagonist role promoting digit joint specification by constraining Wnt activity
Source: Nat Commun. 2026 May 26;17:6835. doi: 10.1038/s41467-026-73549-4 (PMC13389162; doi:10.1038/s41467-026-73549-4)
Supplement: Supplementary file 2 — Peer Review file [file 41467_2026_73549_MOESM2_ESM.pdf]

nature portfolio

Peer Review File

A pivotal Wnt antagonist role promoting digit joint specification by constraining Wnt activity

Corresponding Author: Dr Susan Mackem

This file contains all reviewer reports in order by version, followed by all author rebuttals in order by version.

Attachments originally included by the reviewers as part of their assessment can be found at the end of this file.

Version 0:

Reviewer comments:

Reviewer #1

(Remarks to the Author)
The manuscript entitled “A pivotal role for WNT antagonists in constraining WNT activity to promote interphalangeal joint specification” by Huang et al. investigates the interplay between WNT and BMP signaling pathways in the context of interphalangeal joint formation in 5’Hoxd mutants, using a range of genetic tools and short-term culture assays. The authors build on their previous work (Huang et al., 2015), in which they demonstrated that elevated BMP activity in 5’Hoxd mutants contributes to joint loss, and that this phenotype can be rescued by genetically downregulating BMP signaling. In the current study, they propose that canonical WNT signaling is part of the gene regulatory network involved in joint formation, and that WNT antagonists play a key role in modulating this interaction.
First, the authors use lineage-specific Cre drivers to demonstrate that expression of stabilized β-catenin (activating the canonical WNT pathway) in the interdigit mesenchyme—via Hoxb6CreER—can restore interphalangeal joint formation in 5’Hoxd mutant limb buds, indicating a non-cell-autonomous effect. Through a combination of genetic tools (BMP antagonists, WNT antagonists, and a canonical WNT ligand) and short-term wild-type limb cultures treated with WNT pathway agonists and antagonists, the authors conclude that modulating WNT signaling—either in wild-type tissue or in the context of dysregulated BMP activity (as in 5’Hoxd mutants)—is involved in interphalangeal joint formation by mediating BMP signaling output.
To summarize, the data presented uncover an interesting pathway interconnectivity between BMP and canonical WNT in the context of PFR and interphalangeal joint formation. The findings also show that modulation of WNT signalling by WNT antagonists contributes to this process. The involvement of antagonists reflects well-established roles for BMP and WNT modulators in developmental regulation—and not particularly novel. On the other hand, the identified interaction between the BMP and canonical WNT pathways introduces a potential new node in the regulation of digit joint formation that warrants further investigation. However, without a deeper analysis, the study does as such does not offer a clearly defined or substantive conceptual advance. The study primarily relies on limited developmental stages—namely, late-stage gross skeletal morphology for interdigit analysis and E12.5 pSmad1/5 staining as a readout of BMP activity. Recent studies examining PFR and joint formation in the context of non-canonical WNT, BMP, or Ihh signaling (e.g., Parada et al., 2022; Grall et al., 2024; Leung et al., 2024) have employed more detailed temporal and molecular analyses that may serve as a useful framework for expanding this work.

Major concerns:

(1) The title and framing of the manuscript suggest that (canonical) WNT plays a specification role in interphalangeal joint formation. However, no functional data are provided to support this. The experiments show that activating WNT signaling in a context of elevated BMP activity can rescue joint formation—but this is not equivalent to demonstrating a requirement for canonical WNT in joint specification. Studies show that BMP signaling and WNT5A signaling (non-canonical WNT) are directly involved in digit joint formation (the most recent studies are already cited), but there does not seem to be significant functional (e.g. genetic) evidence that loss of canonical WNT signaling causes joint loss. In this gene regulatory network, it may also be considered that cross-talk occurs between canonical and non-canonical WNT pathways.

(2) The claim that BMP and WNT interact positively rather than antagonistically likely reflects the artificial nature of the experimental conditions, in which either excessive BMP activity or stabilized β-catenin is introduced. Given the extensive

feedback signaling between ligands and their antagonists in both pathways, the differing outcomes observed are more likely a consequence of pathway interconnectivity. This is exemplified by the paradox in which stabilized β-catenin reduces BMP activity in 5'Hoxd mutants, whereas in culture, WNT inhibition leads to loss of Msx2/BMP activity (using the C59 WNT inhibitor), and GSK3 inhibition (a WNT agonist) leads to elevated Msx2/BMP activity.

(3) The pSmad signal used to mark the PFR is often difficult to discern in both control and rescued conditions, making comparisons between experimental groups challenging. This contributes to a fragmented analysis. Incorporating additional markers, such as Activin alongside Gdf5, across multiple stages of digit development would provide a more comprehensive view of the phenotype and give molecular insight into the nature of the rescue phenotypes.

(4) The schemes in Figures 1, 2, 4, and 5 are highly repetitive and appear to be hybrids—aimed at placing the results of the study in a broader context, yet at the same time lacking key components. For example, BMP antagonists are not included, and arrows indicating the connection between WNT ligands and β-catenin are missing. Additionally, the inclusion of FGFs is unclear, as they are not examined in this study.

(5) Figure 5b and e: CHIR 99021 is a well-established WNT agonist, acting as a GSK3 inhibitor. The suggestion that, in this study, it acts directly on BMP-SMAD signaling—as proposed in Fuentealba et al. 2007—is purely speculative. There is no evidence provided here to demonstrate that the observed effects are direct rather than mediated through the WNT pathway, consistent with the interpretation throughout the rest of the study.

(6) Figure S3. This is the only figure that attempts to provide molecular insight based on the transcriptome analysis. Unfortunately, the data are not interpretable due to quality issues. The HCRs shown for the DEGs contain significant background noise (possibly from blood vessels) and, in some cases, are overexposed, particularly in the Sox9 expression images. The quality of these HCRs is insufficient and must be improved to allow for meaningful interpretation.

(7) The discussion should discuss how their findings fit with recent work on PFR and joint formation and how the canonical WNT/BMP interaction would integrate with the other pathways involved in digit joint formation.

Reviewer #2

(Remarks to the Author)
In their manuscript "A pivotal role for Wnt antagonists in constraining Wnt activity to promote digit joint specification", Huang and colleagues report on a series of genetic modifications affecting the Wnt signaling pathway during mouse digit development, and how these impact joint patterning and maturation in the context of an additional engineered genetic variant, namely the removal of 5' Hoxd genes. They use a combination of whole-mount skeletal stains, immunohistochemistry and histology, pharmacological interventions, as well all as transcriptomic data of control and mutant embryos to document the phenotypic consequences of the different modifications.

This should be a paper of broad interest to a readership stretching from general developmental and molecular biologists to specialists in skeletal development and disease.

The data is generally of high quality and I would like to particularly commend the authors for the true genetic 'tour de force' this allelic series represents. I have little to ask in terms of additional experimental work, but would like to see some complementary analyses.

My main issue with the manuscript in its current form, however, is with the interpretation of some of the results – or rather, how the conclusions are phrased. Throughout the text – including the title – the term 'specification' is used rather loosely, without defining whether the authors intend to refer with it to the 'placement' of joint interzones (i.e. patterning), or rather their subsequent 'maturation' into fully functional synovial joints. The schemes in Figs.1,2,4 and 5 strongly suggest that the authors favor the PFR-based digit 'patterning' phase. However, I would argue that most of the data rather suggest that these Wnt signaling pathway manipulations phenotypically affect the joint 'maturation' phase. [The pSMAD levels at the PFR might also be affected, but without multiple sections with proper calibration and quantification, this only seems likely for the RosaWnt3a data in Fig.4k. Related to that, it's difficult to evaluate whether or not PFR-expression of Msx2 is indeed lost in Fig.5d, following Wnt-C59 treatment, although it is clearly abolished in the interdigit areas]. In all other images, I have the impression that the location of the metacarpal-phalangeal joint (MPJ) – and thus the initiation of its interzone – is correctly patterned (see red arrowheads in the attached image, highlighting indentation and/or lighter Alcian blue staining, as well as faint 'late' Gdf5 expression and correctly positioned 'circumferential' Prg4 staining). What is rescued by the Wnt manipulations appears to be a subsequent cartilaginous overgrowth/lack of cavitation in the MPJs of 5'HoxdDel animals. Everything patterning/location-related seems to be determined by the 5'HoxdDel-induced brachydactyly, and not rescued by any of the Wnt manipulations. Accordingly, I suggest to talk about joint "maturation", rather than "specification", and adapt the schemes – i.e. influence of interdigit Wnt antagonists on already initiated joints - and language throughout.

Further suggestions:
- For Figure 1, why are the hindlimbs not shown (at least in the supplement)? Hoxb6-Cre seems to be active in both appendages. Furthermore, given the vast number of embryos produced and photographed, why not capitalize on this impressive qualitative data collection, to quantify/analyze and report the dimensions (length x width, for multiple replciates) of skeletal elements, in the different mutants - at least for the metacarpals and the 'fused' phalanges of digit 3 or 4? It appears that the dimensions change considerably between the genotypes, and having some quantitative 2D-data may also

help to interpret any potential pattering phenotypes, and discuss them in the context of Kavanagh et al. (PNAS 2013) and Grall et al. (PNAS 2024).
- For Figure 3, I would only show PC1 and PC2 in panel b (If the authors prefer their "3D"-visualization, then at least the projections of the different data points onto the PC1/2 plane should be indicated by vertical lines). Since only 145 genes are considered as DE, in panel d rather provide a Z-score normalized heatmap for all 145 genes, showing the 3 replicates of the 3 genotypes together, and highlight/label the 'most relevant' genes. This allows for a much more comprehensive data visualization, and estimation of the noise. The volcano plots could be kept in another figure panel, or placed in the supplement. For panel f, were there any other significant GO terms that could be plotted? A change in Wnt signaling is not particularly surprising, when over-expressing a stabilized version of beta-catenin. (Minor comment: in my Supplementary Table S1 only 8 genes were highlighted in yellow, versus the 9 in Fig.3c?)
- For Figure 4j,k, what are the two 'congruent' holes at the digit tips? Wnt3a was overexpressed genetically, and not delivered via beads, correct?
- For Figure S1a, how reproducible are the elevated Gdf5 expression levels in the perichondrium(?) of 5'HoxdDel animals that are apparent in this image? This might also explain (some) of the cartilaginous overgrowth/joint fusion in 5'HoxdDel animals, given the 'phalanx-inducing' role of Gdf5 suggested by Grall et al.

Minor comments:
- Scale bars are missing throughout. Is it correct to assume that all skeletal prep and histology images have been taken with the same magnification? Please indicate in the figure legends, also for the magnified detailed views, or provide individual scale bars.
- Figure S5: "Bmp activity is not significantly altered during short-term" > maybe use 'substantially', given the lack of quantification/statistical testing?

Reviewer #3

(Remarks to the Author)
In this paper, the authors demonstrate that Wnt and BMP signaling synergize during digit joint formation. This conclusion is supported by a combination of genetic experiments manipulating components of the Wnt and BMP pathways, along with RNA-seq analyses and in vitro assays. Specifically, they show that enhancing canonical Wnt signaling in the interdigital region of 5′Hoxd mutants—which exhibit elevated BMP signaling and defective joint formation—can rescue joint development in a non-cell-autonomous manner. In contrast, increasing Wnt activity within chondrogenic progenitors disrupts their chondrogenic differentiation. Transcriptomic analyses of interdigital tissue from wild-type, mutant, and rescued mutant embryos suggest that secreted Wnt antagonists, such as Dkk2, may mediate these effects.
By analyzing pSmad1/5 levels, the authors show that the rescue of joint formation results from an indirect reduction of BMP activity in the digit tip that requires Dkk2. Based on these findings, they propose a model in which Wnt and FGF signaling cooperate in the digit tip to prevent premature or excessive pSmad1/5 signaling in phalanx progenitors. This regulation would occur through Wnt antagonist–mediated reduction of Wnt signaling, which favors the stabilization of GSK3β. Stabilized GSK3β promotes Smad1/5 linker phosphorylation, leading to their degradation and consequently reduced BMP signaling. The role of GSK3β in modulating pSmad1/5 levels is further supported by in vitro experiments in which Wnt activity is manipulated using small-molecule modulators.
This paper provides relevant information that is likely to have a significant impact on our understanding of digit joint formation. I find the authors' model to be well supported and have no major concerns. Overall, the work is particularly impressive for its comprehensive and precise use of mouse genetics, including multiple conditional mutations.
I would like to raise several points for the authors' consideration, which I hope may help strengthen the clarity the manuscript:
- The authors mention on several occasions that they measure pSmad1/5 levels; however, they only present immunostaining using a specific antibody. They should refer to this more accurately, and, if possible, include some quantification—for instance, by counting the number of positive cells within a defined region of interest, the distal progenitors or the PFR compaction or condensation region. It may be helpful to define the "sub-aer", the region bracketed in Fig 2a-d'. It seems to be limited by the AER to the proximal limit of the marginal sinus.
- Given that the Cre lines used by the authors are expressed throughout most of the autopod tissue, with the exception of the condensations, I would suggest reducing the focus of the genetic manipulations to the interdigits although some of the secreted wnt antagonists seem to be expressed in the proximal interdigit.
- Some of the validation panels for the targets in Figure S3 appear somewhat over-contrasted. Is the secreted antagonist emanating from the proximal interdigit and extending into the distal sub-AER mesoderm? Perhaps this could be addressed or commented on
- While not strictly necessary, I suggest that the authors consider validating their hypothesis regarding GSK3β activity by using antibodies that specifically target the active form of the enzyme in some of their representative mutants.

Minor point:
- It would be desirable for the figures to be self-explanatory, incorporating all relevant information regarding the Cre line used and, when applicable, the timing of tamoxifen administration.
- Please consider switching the order of Figure S2 and Figure S1 to align with the order in which the figures are referenced in the text.
- In the Hoxb6Cre;ROSAWnt3a experiments shown in Figure 4j-k, there appears to be a ventral bias in pSmad activity, was this consistent? Since the immunoanalyses were performed on longitudinal sections, did the authors observe any rescue of the dorsoventral deviation of the ossification centers, as reported by Gonzalez-Martin et al. (PMID: 24930703)?
- Please ensure italics for gene names throughout the manuscript.
- The black and dark blue colors in the PCA are too similar, making it difficult to distinguish between them.

- Please, indicate if variability in phenotypes was observed

Version 1:

Reviewer comments:

Reviewer #2

(Remarks to the Author)
In the revised version of their manuscript, the authors provide an impressive amount of new data and have addressed most of my concerns. I only have a few clarifications and amendments to ask for, before recommending the manuscript for publication:

- In my opinion, it would be beneficial to discuss more explicitly that "only" the MCP is rescued, as opposed to the PIP too. This is important from a developmental as well as an evolutionary perspective, as the MCP delineates skeletal element originating from two distinct modules, i.e. metacarpals and phalanges, and the underlying molecular patterning and maturation mechanisms are likely distinct (see e.g. Bmpr1b phenotype in Soyun et al. 2000, Development) This is also relevant related to the observed and very restricted "time window during which tamoxifen-activated CatCA efficiently restores 5'Hoxd interzone fate is limited to an early stage when digit tips and PFR first appear".
- The novel Figure 3 and Figure S4 are of very high quality, and clearly elevate the overall impact of the manuscript – even though it somewhat reaffirmed my initial impression that joint 'initiation' takes place (albeit delayed, and then "lost"…), and it's the 'maturation' phase that is primarily affected in the rescue (but I guess that is semantics to some extent, and I'm fine with keeping 'specification' throughout). However, I would encourage the authors to at least consider and discuss the idea that the "circumferential ring of Gdf5-expressing cells" might actually play a role in determining joint location within the MCP as well. If indeed this would be the case, that may also explain why the positioning of PIPs scales to the 2D-areas of phalanges, as reported Kavanagh et al. in their PNAS 2013 papers, as the perichondrium can be 'approximated as a sheet of tissue in 2D, rather than 3D.
- Regarding the new Supplementary Figures 6 and 7: One sample of the Hoxd(del/del)/b-catCA appears to be an outlier, along PC1 > it would help the reader to know which samples are which, throughout the paper, and I would encourage the authors to label samples with alphanumerical codes on the different PCA plots (Figures 5b and S6), and use the same codes for the heatmaps in Figure S7.

Reviewer #3

(Remarks to the Author)
The authors have satisfactorily addressed my main concerns.
In response to the other referees' requests, they have added several new experiments that clarify the timing of joint specification rescue and the window of indirect β-Catenin action. These additional data, including the temporal rescue experiments, detailed Gdf5 time course, Sox9 lineage tracing, and signaling analyses, substantially strengthen the manuscript.
I believe the main message of the manuscript that Wnt antagonists promote interzone specification by attenuating canonical Wnt signaling in the subAER progenitor region, thereby increasing GSK3 activity which, in turn, destabilizes BMP signaling is well supported by the multiple genetic manipulations and transcriptome data.
Overall, I find the revised manuscript significantly improved and is now acceptable for publication.

Open Access This Peer Review File is licensed under a Creative Commons Attribution 4.0 International License, which permits use, sharing, adaptation, distribution and reproduction in any medium or format, as long as you give appropriate credit to the original author(s) and the source, provide a link to the Creative Commons license, and indicate if changes were made.
In cases where reviewers are anonymous, credit should be given to 'Anonymous Referee' and the source.
The images or other third party material in this Peer Review File are included in the article's Creative Commons license, unless indicated otherwise in a credit line to the material. If material is not included in the article's Creative Commons license and your intended use is not permitted by statutory regulation or exceeds the permitted use, you will need to obtain permission directly from the copyright holder.
To view a copy of this license, visit https://creativecommons.org/licenses/by/4.0/

We thank all of the reviewers for their thoughtful, detailed comments and constructive criticism that have prompted us to include new experiments and improve the data presentation and clarity of the text. We feel these modifications have greatly improved the manuscript and hope that you will agree.

Reviewer #1:

comments:

.... The authors build on their previous work (Huang et al., 2015), in which they demonstrated that elevated BMP activity in 5'Hoxd mutants contributes to joint loss, and that this phenotype can be rescued by genetically downregulating BMP signaling. In the current study, they propose that canonical WNT signaling is part of the gene regulatory network involved in joint formation, and that WNT antagonists play a key role in modulating this interaction.

The role of Wnts in this context is to inhibit rather than promote joint specification (the conventional view). We realized from the comments of all 3 reviewers that we need to present this point more clearly in the text and have rewritten much of the results section to stress this point.

To summarize, the data presented uncover an interesting pathway interconnectivity between BMP and canonical WNT in the context of PFR and interphalangeal joint formation. The findings also show that modulation of WNT signaling by WNT antagonists contributes to this process. The involvement of antagonists reflects well-established roles for BMP and WNT modulators in developmental regulation—and not particularly novel.

The role of Wnt antagonists as modulators of Wnt signaling is well accepted, however, in this context the role of Wnts (and therefore Wnt antagonists) is unexpected based on previous work on long bone joint formation. Our findings indicate that Wnts have an inhibitory impact on joint formation during initial interzone specification. This differs from the current view of a positive Wnt role promoting joint formation. Consistent with this novel Wnt role, we find that Wnt antagonists promote interzone specification, and propose that this may occur by canonical Wnt ligand inhibition of Gsk3β. Gsk3β has been shown to phosphorylate Smad linkers and destabilize pSmad1/5 in certain contexts (Fuentealba et al, Cell, 2007 and Sapkota et al, Mol. Cell, 2007).

On the other hand, the identified interaction between the BMP and canonical WNT pathways introduces a potential new node in the regulation of digit joint formation that warrants further investigation. However, without a deeper analysis, the study does as such does not offer a clearly defined or substantive conceptual advance. The study primarily relies on limited developmental stages—namely, late-stage gross skeletal morphology for interdigit analysis and E12.5 pSmad1/5 staining as a readout of BMP activity. Recent studies examining PFR and joint formation in the context of non-canonical WNT, BMP, or Ihh signaling (e.g., Parada

et al., 2022; Grall et al., 2024; Leung et al., 2024) have employed more detailed temporal and molecular analyses that may serve as a useful framework for expanding this work.

We agree that a more detailed examination of the timing for rescue of joint specification and the time frame during which β-Catenin acts indirectly (cell non-autonomously) should be determined. We have added several experiments aimed at addressing the timing of rescue and earliest molecular changes, including the time window for efficient joint restoration by recombination of a conditional, stabilized β-Catenin (βCatCA) that behaves as constitutively-active (tamoxifen at E11-E11.5, new Suppl. Figure 1), a detailed time course of first *Gdf5* appearance at the distal digit tip border as each incipient phalanx begins to form in controls, 5'*Hoxd* mutant, and rescued mutant (from E12.5 – E13.75 for MCP through P3, in new Fig. 3, new Suppl. Fig. 4), and we have examined the timing of progenitor commitment to PFR and initiation of new phalanges using Sox9CreER lineage tracing (new Fig. 2) which occurs over a similar time window as that required for tamoxifen activation of βCatCA, and the initial appearance of *Gdf5*+ RNA bands in distal tip border. These experiments all point to ~E12.5 as the earliest time of phenotypic changes in the MCP interzone, the affected joint in the 5'*Hoxd* mutant, at a time when the interzone is induced and just beginning to appear.

Our Sox9 lineage tracing over time (new Figure 2) also shows that new digit phalanges arise from progenitors that are Sox9-negative, (likely subAER), rather than by extension from distal border of existing elements/or PFR, which is very similar to the process in chick. Precocious pSmad1/5 activity is consistently detectable in the subAER when interzone appearance is inhibited as in the 5'*Hoxd* mutant, and is reduced following rescue. Notably, although pSmad1/5 activity in this subAER putative progenitor region varies according to genotype, neither ActivinβA or pSmad3 (also examined, new Suppl. Fig. 5) reached detectable levels in the subAER in any of the genotypes analyzed, and although detected in PFR, were unaltered in the 5'*Hoxd* mutant. Thus we think that early effects on Bmp activity-pSmad1/5 during induction of phalanx/interzone fate play a key role in the 5'*Hoxd* joint phenotype and rescue.

Major concerns:

(1) The title and framing of the manuscript suggest that (canonical) WNT plays a specification role in interphalangeal joint formation. However, no functional data are provided to support this. The experiments show that activating WNT signaling in a context of elevated BMP activity can rescue joint formation—but this is not equivalent to demonstrating a requirement for canonical WNT in joint specification. Studies show that BMP signaling and WNT5A signaling (non-canonical WNT) are directly involved in digit joint formation (the most recent studies are already cited), but there does not seem to be significant functional (e.g. genetic) evidence that loss of canonical WNT signaling causes joint loss. In this gene regulatory network, it may also be considered that cross-talk occurs between canonical and non-canonical WNT pathways.

As mentioned above, we are not proposing a role for canonical Wnts, but for Wnt antagonists to maintain plasticity and enable interzone specification by keeping canonical Wnt ligand activity in check in the subAER progenitor region. This finding is indeed unexpected, but is supported both by our transcriptome analysis and by the results of different genetic perturbations that we evaluated. This analysis pointed to *Dkk2* as a candidate secreted factor induced by activated βCatCA in interdigits that plays a role in restoring 5'*Hoxd* mutant joint formation. Although canonical and non-canonical WNT pathways compete with each other for shared effectors and the non-canonical pathway has a clear role in digit phalanx formation, there is no definite evidence that it also regulates interzones/joints. The recent study of Parada et al (Dev. Cell, 2022) does not focus on joint formation, but rather on first phalanx/condensation induction and mechanical influences. In their model, the Wnt5a KO fails to produce appropriate mechanical shape changes to drive condensation and consequently phalanx formation fails altogether; they propose that the Wnt5a KO forms no phalanges at all. Consistent with this model, mutants in noncanonical signaling with less severe phenotypes (eg. Wnt5a receptor ROR2; Witte et al, 2010) have reduced phalanx numbers, but joint formation per se is not affected (based both on *Gdf5* expression and late skeletal digit phenotypes).

(2) The claim that BMP and WNT interact positively rather than antagonistically likely reflects the artificial nature of the experimental conditions, in which either excessive BMP activity or stabilized β-catenin is introduced. Given the extensive feedback signaling between ligands and their antagonists in both pathways, the differing outcomes observed are more likely a consequence of pathway interconnectivity. This is exemplified by the paradox in which stabilized β-catenin reduces BMP activity in 5'Hoxd mutants, whereas in culture, WNT inhibition leads to loss of Msx2/BMP activity (using the C59 WNT inhibitor), and GSK3 inhibition (a WNT agonist) leads to elevated Msx2/BMP activity.

There were no experiments in which excess-(GOF) Bmp was introduced via engineering. Endogenous Bmps are naturally elevated in the 5'*Hoxd* mutant. Stabilized βCatenin was not expressed in the digit tip region, only in the interdigits and acted indirectly (cell non-autonomously) to restore joint formation. Expression of Wnt3a ligand in the digit tip region did not produce a decrease in Bmp activity, but elevated it (increased pSmad1/5; see new Figure 6k) and this subsequently led to joint loss in digits of control embryos. The culture and in vivo results are in perfect agreement with each other - there is no paradox. We think that this mis-conception again reflects poor communication/clarity on our part in presenting the results, which we have attempted to correct in the revised text. In the context of the rescue in vivo, it is not activated βCatenin, but its downstream induced, secreted targets (Wnt antagonists), that act cell non-autonomously on digit tips to reduce Bmp activity. We have attempted to rectify this lack of clarity on our part throughout the text. We also note in the text (pg. 13-14) that in the above contexts, Wnt ligand and activated βCatenin differ in functioning either upstream vs downstream of the destruction complex (including Gsk3β).

(3) The pSmad signal used to mark the PFR is often difficult to discern in both control and

rescued conditions, making comparisons between experimental groups challenging. This contributes to a fragmented analysis. Incorporating additional markers, such as Activin alongside Gdf5, across multiple stages of digit development would provide a more comprehensive view of the phenotype and give molecular insight into the nature of the rescue phenotypes.

We agree and, as mentioned above, we examined pSmad3 levels, and *Activinβ A*, which are thought to act upstream of pSmad1/5 induction in the PFR and these were unchanged from controls in both the 5'*Hoxd* mutant and following rescue (now included in new Suppl. Figure 5). We also showed that early progenitors for PFR do not arise from Sox9+ distal cells or existing PFR, but from a new uncommitted progenitor population, likely in the subAER region as occurs in chick (new Figure 2). This subAER region displays differences in pSmad1/5 levels in both the mutant and rescue experiments, which is relevant and is in some cases more obvious than PFR pSmad changes and this is now highlighted in the figures. Our results suggest that targeting of the subAER progenitors as they transit into nascent PFR alters their ability to adopt either interzone or phalanx fate. We also noticed that neither *Activinβ A* or pSmad3 are significantly expressed in the subAER region. For all of these reasons we feel that the pSmad1/5 changes in the subAER represents the earliest phenotypic change, consistent with the time course of *Gdf5* induction across stages in controls vs mutant. This would occur early, at the time that interzones/phalanges are first being sequentially specified, which our *Gdf5* time course indicates is at about E12.5 for the MCP interzone that is typically lost in the 5'*Hoxd* mutant (new Figure 3, Suppl. Fig. 4).

(4) The schemes in Figures 1, 2, 4, and 5 are highly repetitive and appear to be hybrids—aimed at placing the results of the study in a broader context, yet at the same time lacking key components. For example, BMP antagonists are not included, and arrows indicating the connection between WNT ligands and β-catenin are missing. Additionally, the inclusion of FGFs is unclear, as they are not examined in this study.

These were NOT intended to add a 'broader context' or to present a comprehensive summary of pathway components, but only to indicate what preliminary conclusions could be gleaned from the results presented in the figure summarized by the schematic, and what the next step/question to consider might be. Hence, they may seem repetitive because they build on the findings summarized in the previous figure. Since much of the genetics becomes complex, particularly with indirect, cell non-autonomous effects to keep in mind, we thought this would be helpful for a general reader who may not be intimately familiar with the pathways. We have simplified these schematics to show mainly what could be concluded at each stage from successive experimental questions to make it clearer this is not intended as a comprehensive summary of the pathway regulatory networks.

(5) Figure 5b and e: CHIR 99021 is a well-established WNT agonist, acting as a GSK3 inhibitor. The suggestion that, in this study, it acts directly on BMP-SMAD signaling—as proposed in Fuentealba et al. 2007—is purely speculative. There is no evidence provided

here to demonstrate that the observed effects are direct rather than mediated through the WNT pathway, consistent with the interpretation throughout the rest of the study.

If going through the Wnt pathway, these effects would still be direct. We think that Wnt ligand-receptor binding IS acting through its normal target (Gsk3β). Gsk3β has been implicated in pSmad1 degradation via linker phosphorylation and Wnt ligands inhibit Gsk3β as part of the Wnt destruction complex. To indicate that this possible mechanism is not proven to operate in the present context, we have modified the results and discussion to indicate that it is a proposed model and is consequently hypothetical at present.

(6) Figure S3. This is the only figure that attempts to provide molecular insight based on the transcriptome analysis. Unfortunately, the data are not interpretable due to quality issues. The HCRs shown for the DEGs contain significant background noise (possibly from blood vessels) and, in some cases, are overexposed, particularly in the Sox9 expression images. The quality of these HCRs is insufficient and must be improved to allow for meaningful interpretation.

We agree that the original Figure S3 HCR analysis could be improved and we have replaced this figure with better images of relevant DEGs that were examined (new Suppl. Figure 8). This is not the only figure attempting to provide insight. Examination of whether *Dkk2* played any role, as predicted from the transcriptome analysis, was performed and showed that loss of *Dkk2* in the 5'*Hoxd* mutant does contribute to the phenotype. Introducing the *Dkk2*-KO abolished the rescue by interdigit activated βCatenin (now Figure 6), indicating that this induced target (which is also normally expressed in interdigits and is substantially reduced in the 5'*Hoxd* mutant) does contribute to the rescue.

(7) The discussion should discuss how their findings fit with recent work on PFR and joint formation and how the canonical WNT/BMP interaction would integrate with the other pathways involved in digit joint formation.

We have commented on the role of Wnt5a and why we don't think that our results would be explained by effects of the canonical pathway on non-canonical activity in the Discussion. (Wnt5a impacts formation of the first phalanx and on subsequent phalanges since the mutant fails to make any phalanges, but less severe phenotypes as seen in the Wnt5a receptor *ROR2* KO, do not have any loss of joint formation although they do form fewer phalanges).

Although the *Ihh* mutant certainly does have phalangeal and joint formation abnormalities and *Ihh* has been proposed having a function in recruiting progenitors for digit growth (Koyama et al, Annal NY Acad Sci., 2007; Witte et al, 2009; Gao et al, Nature, 2009), the onset of *Ihh* expression in digit tips at about E13 and the analyses of timing of *Ihh* mutant phenotypic changes, suggest that *Ihh* plays a critical role at a somewhat later stage (~E13-13.5), downstream of the effects described in this report that are initiated between E11.5-

12.5 and more likely affecting established PFR/phalanx anlage than progenitors. We have also noted this in the Discussion.

Reviewer #2:

Comments:

This should be a paper of broad interest to a readership stretching from general developmental and molecular biologists to specialists in skeletal development and disease.

The data is generally of high quality and I would like to particularly commend the authors for the true genetic 'tour de force' this allelic series represents. I have little to ask in terms of additional experimental work, but would like to see some complementary analyses.

We thank the reviewer for their supportive and constructive comments.

My main issue with the manuscript in its current form, however, is with the interpretation of some of the results – or rather, how the conclusions are phrased. Throughout the text – including the title – the term 'specification' is used rather loosely, without defining whether the authors intend to refer with it to the 'placement' of joint interzones (i.e. patterning), or rather their subsequent 'maturation' into fully functional synovial joints. The schemes in Figs.1,2,4 and 5 strongly suggest that the authors favor the PFR-based digit 'patterning' phase. However, I would argue that most of the data rather suggest that these Wnt signaling pathway manipulations phenotypically affect the joint 'maturation' phase. [The pSMAD levels at the PFR might also be affected, but without multiple sections with proper calibration and quantification, this only seems likely for the RosaWnt3a data in Fig.4k.

Based on additional data in the revised manuscript, showing that digit progenitors arise from uncommitted cells (likely in the subAER region) and not by extension from Sox9+ cells (distal existing element or PFR, see new Fig. 2) and a detailed time course documenting the first appearance of successive *Gdf5*+ interzones at the digit tip border in controls, 5'*Hoxd* mutant and rescued mutant (new Fig. 3, and S4), we feel that the first phenotypic change is already evident at the time of incipient interzone formation (E12.5 for the MCP interzone). The early time interval necessary for effective tamoxifen induced βCatCA activation (ending after E11.5 treatment, new Fig. S1) also suggests that an early step in phalanx/interzone induction is affected by the rescue ("patterning" phase).

The above evidence also suggests that the process in highly conserved with chick, and likely that progenitors arise in the subAER region. This region is thickened and shows considerably, and consistently higher pSmad1/5 activity in the 5'*Hoxd* mutant, or following RosaWnt3a misexpression, or in contexts where rescue of the 5'*Hoxd* mutant is abolished (reduced *Noggin* or *Dkk2* KO). Because the subAER region is very small in mouse, we think quantitative comparisons would be difficult to perform with good statistical power and are

less essential, since the qualitative differences are more striking in this region. It should be noted that for each experiment, different genotypes collected were examined together as a group and marked and co-stained together with the same antibody dilution, (or co-hybridized and analyzed by HCR), so the intensity comparisons across embryo samples shown for a particular figure are valid, albeit only at a qualitative level. This is now stated in the methods.

Related to that, it's difficult to evaluate whether or not PFR-expression of Msx2 is indeed lost in Fig.5d, following Wnt-C59 treatment, although it is clearly abolished in the interdigit areas]. In all other images, I have the impression that the location of the metacarpal-phalangeal joint (MPJ) – and thus the initiation of its interzone – is correctly patterned (see red arrowheads in the attached image, highlighting indentation and/or lighter Alcian blue staining, as well as faint 'late' Gdf5 expression and correctly positioned 'circumferential' Prg4 staining). What is rescued by the Wnt manipulations appears to be a subsequent cartilaginous overgrowth/lack of cavitation in the MPJs of 5'HoxdDel animals. Everything patterning/location-related seems to be determined by the 5'HoxdDel-induced brachydactyly, and not rescued by any of the Wnt manipulations. Accordingly, I suggest to talk about joint "maturation", rather than "specification", and adapt the schemes – i.e. influence of interdigit Wnt antagonists on already initiated joints - and language throughout.

In the short-term limb bud culture, the Msx2 stained region is restricted at the distal digit tip tissue adjacent to AER in control. Unlike up-regulation by CHIR, this makes the down-regulation of Msx2 signaling difficult to appreciate in the Wnt-C59 treated culture. Therefore, we also examined pSmad1/5 staining to evaluate BMP activity changes directly for both CHIR and Wnt-C59 (now Figure 7d). pSmad1/5 is clearly reduced following Wnt-C59 treatment.

Our temporal analysis of *Gdf5* first appearance and time window for tamoxifen efficacy, as summarized above, indicate that early events during interzone induction are affected. We have noted in the text results (page 8-9) and new Figures 3, Suppl. S4, the residual circumferential *Gdf5* expression seen in the 5'*Hoxd* mutant, which is usually transient and variable. This external *Gdf5*+ ring is also seen in other mutants thought to impact early interzone formation, for eg. *Creb5* (Zhang, PNAS, 2025) and *Noggin* mutants (Brunet, Science, 1998) as well as *Ihh* null digits (Koyama, Ann NY Acad Sci, 2007), and may reflect a separate regulation of perichondrial/mesenchymal interdigit *Gdf5*, that is still properly patterned independently of the interzone per se. We have now commented on this in the results. The indentation is very superficial and is not invariably seen. For example, in the red arrows of attached pdf sent along with review, digit 4 does not have an appreciable indentation. We have also never observed any frank, easily appreciated 'cavitation' within the presumptive interzone region itself, at earlier or at later stages in the 5'*Hoxd* mutant.

Further suggestions:

- For Figure 1, why are the hindlimbs not shown (at least in the supplement)? Hoxb6-Cre

seems to be active in both appendages. Furthermore, given the vast number of embryos produced and photographed, why not capitalize on this impressive qualitative data collection, to quantify/analyze and report the dimensions (length x width, for multiple replciates) of skeletal elements, in the different mutants - at least for the metacarpals and the 'fused' phalanges of digit 3 or 4? It appears that the dimensions change considerably between the genotypes, and having some quantitative 2D-data may also help to interpret any potential pattering phenotypes, and discuss them in the context of Kavanagh et al. (PNAS 2013) and Grall et al. (PNAS 2024).

The hindlimb phenotypes are different; both joints and phalanges are considerably less affected. In the forelimb, joints are never formed and then fuse, and as detailed above, several lines of evidence indicate that phenotypic changes begin very early. The later growth/growth plates etc. may very well also be affected, but may reflect the effects of multiple variables at different stages, and may not necessarily be related only to early interzone specification phenotypes. While the morphometric analysis suggested may be interesting to follow up on, we think it is not part of the main focus of the current report and would be more appropriately studied independently.

- For Figure 3, I would only show PC1 and PC2 in panel b (If the authors prefer their "3D"-visualization, then at least the projections of the different data points onto the PC1/2 plane should be indicated by vertical lines). Since only 145 genes are considered as DE, in panel d rather provide a Z-score normalized heatmap for all 145 genes, showing the 3 replicates of the 3 genotypes together, and highlight/label the 'most relevant' genes. This allows for a much more comprehensive data visualization, and estimation of the noise. The volcano plots could be kept in another figure panel, or placed in the supplement. For panel f, were there any other significant GO terms that could be plotted? A change in Wnt signaling is not particularly surprising, when over-expressing a stabilized version of beta-catenin. (Minor comment: in my Supplementary Table S1 only 8 genes were highlighted in yellow, versus the 9 in Fig.3c?)

We thank the reviewer for pointing out the arithmetic error in the Venn diagram - the overlap is indeed 8 genes - and this has now been corrected (now Figure 5).

In the present case, the 3rd principal component is contributory to differences between the 5'*Hoxd* mutant and rescued mutant genotypes, which are considerably fewer than between 5'*Hoxd* mutant and control 5'*Hoxd*$^{+/\Delta}$. To demonstrate this more clearly, we have added 2D PC maps comparing each of the 1st three PCs in new Suppl. Figure 6 to show the impact of each PC (1-3) in pairwise combinations.

The volcano plots show both Padj and FC so that the significance of DE values is clear whereas a Z-score heatmap of 145 DEs for all 3 genotypes would not. We have expanded the relevant scale so these plots are easier to evaluate. Heatmaps of the normalized expression for each of the 3 biological replicates for each genotype have now also been added as new Supplemental Fig. 7, in pairwise comparisons of genes with a significant Padj.

Since most of the DEGs don't overlap, we prefer to show the normalized expression heatmaps in pairwise comparisons instead, including only genes with significant differences for each comparison. These were each divided into 2 groups with low vs. high expression levels so that visual color differences can be appreciated more easily between replicate samples and between different genotypes. This preserves both significance and allows the reader to readily judge the degree of variability in expression between replicates for each genotype in the DEGs. It should be noted that all of this information, including individual replicate counts, is also provided in Suppl. Data set 2 (formerly Table S2), albeit not in a simple visual manner.

We agree about the comment regarding GO term enrichment and Wnt target changes which would be expected in the rescue, and these were the major significant changes observed, which were substantially fewer between 5'*Hoxd* mutant and rescue, than 5'*Hoxd* mutant vs control. However, our subsequent analysis indicated that these differences were indeed responsible for the rescue. In fact, several significant Wnt pathway changes were also identified in the 5'*Hoxd* mutant vs control (7 out of 57 DEs were in Wnt pathway components or targets). This first led us to suspect that the Wnt pathway changes might actually be involved mechanistically in the βCatCA rescue.

- For Figure 4j,k, what are the two 'congruent' holes at the digit tips? Wnt3a was overexpressed genetically, and not delivered via beads, correct?

This is the marginal sinus (created by expanded capillary network in the subAER region), now annotated in control image and noted in the legend (new Figure 4) for the first figure showing digit tip pSmad1/5 at E12.5 -13.5 stages. Wnt3a was overexpressed conditionally from a Rosa locus transgene, indicated in legend (now Figure 6) and in Methods.

- For Figure S1a, how reproducible are the elevated Gdf5 expression levels in the perichondrium(?) of 5'HoxdDel animals that are apparent in this image? This might also explain (some) of the cartilaginous overgrowth/joint fusion in 5'HoxdDel animals, given the 'phalanx-inducing' role of Gdf5 suggested by Grall et al.

The *Gdf5* marked MCP interzone is lost in 5'Hoxd mutant. As detailed above, circumferential *Gdf5* expression is seen in mesenchymal interdigit/perichondrial cells adjacent to sites where interzones fail to form in the digits in several mutants, in addition to 5'*Hoxd*, including *Creb5*, *Noggin*, *Ihh*, which is now noted in the text. The circumferential perichondrial expression of *Gdf5* in 5'*Hoxd* mutant MCP is reproducibly present early, but usually disappears by the time all phalangeal primordia appear, never extends internally to suggest any partial interzone formation, and never persists to mature cavitation stages with subsequent "fusion".

The trace level of *Gdf5* in d4 in panel **a** of what is now Suppl. Figure 3, reflects some variability in the phenotype noted in the revised methods (digit 3 is always lost and most severely affected, then d4; d2 and d5 were more variable, so our analysis generally focused on d3,

d4). Usually *Gdf5* is limited to and external ring of expression and is transient, when seen. The other panel with arrow shows *Prg4* expression, which like *Gdf5*, may be circumferentially expressed, but at a much later stage. Not very many mice were examined postnatally, because, although the 5'*Hoxd* mutant mice can survive into adulthood, the βCatCA -rescued mice do not survive postnatally beyond the immediate perinatal period.

Minor comments:

- Scale bars are missing throughout. Is it correct to assume that all skeletal prep and histology images have been taken with the same magnification? Please indicate in the figure legends, also for the magnified detailed views, or provide individual scale bars.

All panels for particular image type (HCR, IF, skeletal) within panels of a figure were generally at the same magnification. Scale bars have now been added to all figures to indicate magnification.

- Figure S5: "Bmp activity is not significantly altered during short-term" > maybe use 'substantially', given the lack of quantification/statistical testing?

we agree; this has been changed. (in Figure S5, which is now Figure S10).

Reviewer #3:

comments:

...This paper provides relevant information that is likely to have a significant impact on our understanding of digit joint formation. I find the authors' model to be well supported and have no major concerns. Overall, the work is particularly impressive for its comprehensive and precise use of mouse genetics, including multiple conditional mutations.

We thank reviewer for supportive comments and constructive criticism, suggestions.

I would like to raise several points for the authors' consideration, which I hope may help strengthen the clarity the manuscript:

- The authors mention on several occasions that they measure pSmad1/5 levels; however, they only present immunostaining using a specific antibody. They should refer to this more accurately, and, if possible, include some quantification—for instance, by counting the number of positive cells within a defined region of interest, the distal progenitors or the PFR compaction or condensation region. It may be helpful to define the "sub-aer", the region bracketed in Fig 2a-d'. It seems to be limited by the AER to the proximal limit of the marginal sinus.

We agree that to define the region of interest in the figure could make the model clearer.

Indeed, we have modified the brackets so that the sub-AER region is more completely seen and added annotations-explanations in the first immuno-stained figure (now Figure 4). pSmad1/5 detection in sub-AER changes prominently between the different genotypes. The sub-AER region (between ectoderm and marginal sinus) is very attenuated in mouse with small cell numbers, and is difficult to quantitate with sufficient numbers to achieve a reasonable statistical power. However, we feel that the sub-AER changes, which are very consistent for a given genotype, are qualitatively clear. We have attempted to qualify statements about pSmad levels to indicate that precise quantification of levels has not been performed.

- Given that the Cre lines used by the authors are expressed throughout most of the autopod tissue, with the exception of the condensations, I would suggest reducing the focus of the genetic manipulations to the interdigits although some of the secreted wnt antagonists seem to be expressed in the proximal interdigit.

The Hoxb6CreER line is broadly expressed in noncondensed limb mesenchyme, but activation of βCatCA in other tissues aside from interdigit, specifically in cartilage, does not rescue interzone formation, whereas activation by a Cre transgene very selectively expressed in interdigits (OsrCre), does also rescue interzone/joint formation, albeit with more limited expression and late timing. Because of this, we think it likely that the interdigit expression is the critical site for the rescue.

- Some of the validation panels for the targets in Figure S3 appear somewhat over-contrasted. Is the secreted antagonist emanating from the proximal interdigit and extending into the distal sub-AER mesoderm? Perhaps this could be addressed or commented on

Some of the expression of candidate βCatCA-activation targets is indeed more proximal (also seen in the survey of Wnt pathway expression in interdigits shown in suppl. figures of Raspopovic et al (Science, 2014) and apparently diffuses or perhaps is transported via cellular projections (cytonemes). The demonstration of a role for *Dkk2*, which is expressed proximally in interdigits, is dependent on 5'*Hoxd* function for expression and required for rescue of the 5'*Hoxd* mutant strongly suggests that the effect of the secreted antagonist can extend long-range. We agree that the quality of the validated expression examples could be improved and have now been replaced the HCR images with more clear examples in the new Figure S8.

- While not strictly necessary, I suggest that the authors consider validating their hypothesis regarding GSK3β activity by using antibodies that specifically target the active form of the enzyme in some of their representative mutants.

This is an excellent suggestion; we attempted to do this, but were unable to find antibodies that gave clear and strong reactivity in IF. We have made the speculative nature of our observation more clear by now referring to our conclusion as a proposed model for the mechanism by which Wnt signaling cooperates with Bmps to enhance pSmad1/5 detection.

Minor point:

- It would be desirable for the figures to be self-explanatory, incorporating all relevant information regarding the Cre line used and, when applicable, the timing of tamoxifen administration.

We have now included this information in each legend and in the text.

- Please consider switching the order of Figure S2 and Figure S1 to align with the order in which the figures are referenced in the text.

We have done this in the revised manuscript; all the figures are now numbered according to the order in which they are first cited .

- In the Hoxb6Cre;ROSAWnt3a experiments shown in Figure 4j-k, there appears to be a ventral bias in pSmad activity, was this consistent? Since the immunoanalyses were performed on longitudinal sections, did the authors observe any rescue of the dorsoventral deviation of the ossification centers, as reported by Gonzalez-Martin et al. (PMID: 24930703)?

This is an interesting point, but unfortunately, no precise tracking of DV orientation was applied, since this did not appear to be directly relevant to the question being addressed. Our rough impression was that there is no consistent bias. Whether a bias in ossification was corrected by the βCatCA rescue is difficult to evaluate with the Hoxb6CreER-activated rescue by βCatCA because ossification is delayed and the pups also don't survive beyond the perinatal period owing to βCatCA expression at other sites. In the case of OsrCre, with more limited activation of βCatCA expression, the deviation of ossification could be assessed in some skeletal analyses, but correction of deviation was variable and not consistently observed.

- Please ensure italics for gene names throughout the manuscript. This has been done.

- The black and dark blue colors in the PCA are too similar, making it difficult to distinguish between them.

We have replaced the black by a lighter gray tone so that each color now appears more unique.

- Please, indicate if variability in phenotypes was observed.

The 5'*Hoxd* mutant was on an inbred FVB background, and phenotypes were very consistent in mutant and rescued mutant. Interzone/joint formation in digit 2 was largely preserved in the mutant, but was occasionally affected (~ 17% MCP loss in d2 and 32% loss in d5 in

5'*Hoxd* mutant, reported in Huang et al, 2016). In 5'*Hoxd*$^{+/\Delta}$ controls, d5 was rarely biphalangeal (approx. 5%) but no other abnormal phenotypes were observed. Our analyses of interzone rescue focused on d3, d4 since these were most consistently abnormal in the 5'*Hoxd* mutant and MCP restoration in rescue by βCatCA activation approached 100%. A comment regarding the level of phenotypic variability has been added in the methods section describing mouse strains used.

RESPONSES TO REVIEWERS' COMMENTS

Responses to Reviewer #2 (Remarks to the Author):

In the revised version of their manuscript, the authors provide an impressive amount of new data and have addressed most of my concerns. I only have a few clarifications and amendments to ask for, before recommending the manuscript for publication:

- In my opinion, it would be beneficial to discuss more explicitly that "only" the MCP is rescued, as opposed to the PIP too. This is important from a developmental as well as an evolutionary perspective, as the MCP delineates skeletal element originating from two distinct modules, i.e. metacarpals and phalanges, and the underlying molecular patterning and maturation mechanisms are likely distinct (see e.g. Bmpr1b phenotype in Soyun et al. 2000, Development) This is also relevant related to the observed and very restricted "time window during which tamoxifen-activated CatCA efficiently restores 5'Hoxd interzone fate is limited to an early stage when digit tips and PFR first appear".

We feel that the inability to restore the PIP joints is a result of absence of the entire middle phalanx (P2), which is not restored by the CatCA rescue. The absence of the middle phalanx is seen in other contexts, for example in the compound 5'*Hoxd*/Gli3 mutant which has complete restoration of MCP joints in biphalangeal digits (P2 missing). In yet other contexts also previously evaluated, when both Bmp ligand availability and receptor dosage are genetically reduced in 5'*Hoxd* mutant, triphalangeal digits with <u>both</u> P2 and MCP and PIP joints can be restored. We also note that a PIP circumferential *Gdf5*+ ring is not seen at any time in the 5'*Hoxd* mutant digits even though the DIP *Gdf5* band subsequently appears with normal timing, and this is most consistent with a total absence of the 2nd (middle) phalanx. Consequently, we don't think that the restoration is related to a distinctive origin for the MCP. We have added this point to the discussion.

- The novel Figure 3 and Figure S4 are of very high quality, and clearly elevate the overall impact of the manuscript – even though it somewhat reaffirmed my initial impression that joint 'initiation' takes place (albeit delayed, and then "lost"...), and it's the 'maturation' phase that is primarily affected in the rescue (but I guess that is semantics to some extent, and I'm fine with keeping 'specification' throughout). However, I would encourage the authors to at least consider and discuss the idea that the "circumferential ring of Gdf5-expressing cells" might actually play a role in determining joint location within the MCP as well. If indeed this would be the case, that may also explain why the positioning of PIPs scales to the 2D-areas of phalanges, as reported Kavanagh et al. in their PNAS 2013 papers, as the perichondrium can be 'approximated as a sheet of tissue in 2D, rather than 3D.

Yes it is possible that the circumferential *Gdf5* expression provides a positional cue, perhaps for proper ligament positioning around joints, that is not lost even when interzone specification in pre-PFR cells is absent. However, it is unclear whether this *Gdf5*+ population is a separately induced and regulated population, which would explain it's

persistence even when interzone induction is absent. We feel that the early timing of expression changes in the 5'*Hoxd* mutant and the mutant rescue by CatCA argue that early induction rather than subsequent joint maturation is impacted. These points are now added to the discussion.

- Regarding the new Supplementary Figures 6 and 7: One sample of the Hoxd(del/del)/b-catCA appears to be an outlier, along PC1 > it would help the reader to know which samples are which, throughout the paper, and I would encourage the authors to label samples with alphanumerical codes on the different PCA plots (Figures 5b and S6), and use the same codes for the heatmaps in Figure S7.

Yes, for one sample of the 2nd biological replicate (mutant rescue by βCA), the NCI Genomics Core had a technical problem during library preparation that necessitated re-generating and sequencing separately, the library derived from the cDNA of that sample, creating a "batch effect". All independent samples for each of the 3 genotype are consistently labeled as 1,2,3 (control, 5'Hoxd mutant, βCA-rescued mutant) according to the replicate labeling used in both Supplementary data sets 1 and 2 (DEseq2 analysis) and in the raw data files deposited with GEO (GSE317957). For the βCA replicate 2 that was sequenced separately, an asterisk is added (2*) to indicate this as the "outlier". This information is now detailed in the supplementary information provided in the supplementary pdf file, which is referred to in the legends of Figure 5b, and Supplementary Figures 6 and 7, for which the same 1, 2 (or 2*), 3 labels are shown in the PCAs and heatmaps to identify specific replicates.

Reviewer #3 (Remarks to the Author): no changes suggested.

The authors have satisfactorily addressed my main concerns.
In response to the other referees' requests, they have added several new experiments that clarify the timing of joint specification rescue and the window of indirect β-Catenin action. These additional data, including the temporal rescue experiments, detailed Gdf5 time course, Sox9 lineage tracing, and signaling analyses, substantially strengthen the manuscript.
I believe the main message of the manuscript that Wnt antagonists promote interzone specification by attenuating canonical Wnt signaling in the subAER progenitor region, thereby increasing GSK3 activity which, in turn, destabilizes BMP signaling is well supported by the multiple genetic manipulations and transcriptome data.
Overall, I find the revised manuscript significantly improved and is now acceptable for publication.

Reviewer #1: no comments provided.

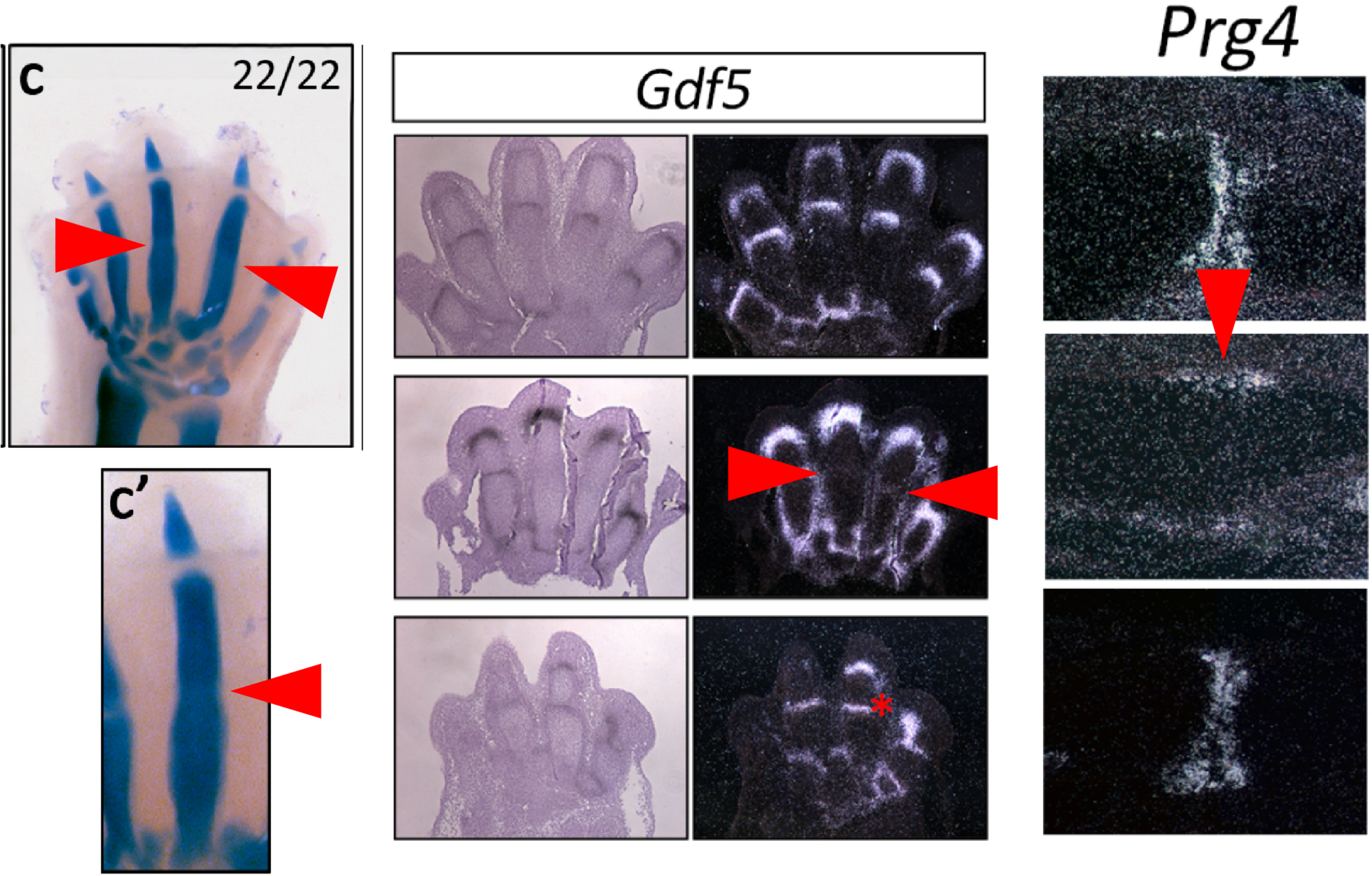
c
22/22
c'
Gdf5
Prg4
*
